# High-flow nasal cannula versus non-invasive ventilation for acute hypercapnic respiratory failure in adults: a systematic review and meta-analysis of randomized trials

**DOI:** 10.1186/s13054-022-04218-3

**Published:** 2022-11-09

**Authors:** N. Ovtcharenko, E. Ho, W. Alhazzani, A. Cortegiani, B. Ergan, R. Scala, G. Sotgiu, D. Chaudhuri, S. Oczkowski, K. Lewis

**Affiliations:** 1 Department of Medicine, McMaster University, 1280 Main Street West, Hamilton, ON L8S 4L8 Canada; 2 Faculty of Health Sciences, McMaster University, Hamilton, ON Canada; 3 Department of Health Research Methods, Evidence, and Impact, Hamilton, ON Canada; 4 Department of Surgical Oncological and Oral Science (Di.Chir.On.S), University of Palermo, Palermo, Italy; 5 Department of Anesthesia Intensive Care and Emergency, Policlinico Paolo Giaccone, Palermo, Italy; 6 Department of Pulmonary and Critical Care, Dokuz Eylul University School of Medicine, Izmir, Turkey; 7 Pulmonology and Respiratory Intensive Care Unit, Cardio-Thoraco-Neuro-Vascular Department, USL Toscana Sudest, S Donato Hospital, Arezzo, Italy; 8 Clinical Epidemiology and Medical Statistics Unit, Department of Medicine, Surgery and Pharmacy, University of Sassari, Sassari, Italy

**Keywords:** Non-invasive ventilation, High-flow nasal cannula, Hypercapnic respiratory failure

## Abstract

**Background:**

Non-invasive ventilation (NIV) with bi-level positive pressure ventilation is a first-line intervention for selected patients with acute hypercapnic respiratory failure. Compared to conventional oxygen therapy, NIV may reduce endotracheal intubation, death, and intensive care unit length of stay (LOS), but its use is often limited by patient tolerance and treatment failure. High-flow nasal cannula (HFNC) is a potential alternative treatment in this patient population and may be better tolerated.

**Research question:**

For patients presenting with acute hypercapnic respiratory failure, is HFNC an effective alternative to NIV in reducing the need for intubation?

**Methods:**

We searched EMBASE, MEDLINE, and the Cochrane library from database inception through to October 2021 for randomized clinical trials (RCT) of adults with acute hypercapnic respiratory failure assigned to receive HFNC or NIV. The Cochrane risk-of-bias tool for randomized trials was used to assess risk of bias. We calculated pooled relative risks (RR) for dichotomous outcomes and mean differences (MD) for continuous outcomes, with corresponding 95% confidence intervals (CI) using a random-effects model.

**Results:**

We included eight RCTs (*n* = 528) in the final analysis. The use of HFNC compared to NIV did not reduce the risk of our primary outcome of mortality (RR 0.86, 95% CI 0.48–1.56, low certainty), or our secondary outcomes including endotracheal intubation (RR 0.80, 95% CI 0.46–1.39, low certainty), or hospital LOS (MD − 0.82 days, 95% CI − 1.83–0.20, high certainty). There was no difference in change in partial pressure of carbon dioxide between groups (MD − 1.87 mmHg, 95% CI − 5.34–1.60, moderate certainty).

**Interpretation:**

The current body of evidence is limited in determining whether HFNC may be either superior, inferior, or equivalent to NIV for patients with acute hypercapnic respiratory failure given imprecision and study heterogeneity. Further studies are needed to better understand the effect of HFNC on this population.

**Supplementary Information:**

The online version contains supplementary material available at 10.1186/s13054-022-04218-3.

## Background

Non-invasive positive pressure ventilation (NIV) delivers two levels of pressure during the respiratory cycle—a lower pressure during the expiratory phase and a higher pressure during the inspiratory phase. The pressure differential assists with the washout of accumulated carbon dioxide (CO_2_) and supports respiratory muscles to reduce work of breathing [1]. As such, NIV has been found to reduce mortality and need for intubation in patients with acute hypercapnic respiratory failure secondary to acute exacerbation of chronic obstructive pulmonary disease (AECOPD) [2, 3], NIV is also suggested for use in acute respiratory failure in immunocompromised and postoperative patients, and for prevention of post-extubation respiratory failure in high-risk patients [2].

Despite wide potential for application, NIV use can be limited due to patient intolerance of the interface or positive pressure. NIV requires a tight-fitting mask or helmet, delivery of high pressures to an awake patient, is associated with skin breakdown after prolonged use, causes gastric insufflation with increased risk of aspiration, can be associated with patient-ventilator asynchrony, and limits both secretion management and nutritional intake [4, 5]. Patients who cannot tolerate NIV will often require invasive mechanical ventilation [6–8].

High-flow nasal cannula (HFNC) is an oxygen delivery device which utilizes high inspiratory flows of up to 60L/min through a nasal cannula to deliver up to 100% fraction of inspired oxygen (FiO_2_). HFNC has been studied in the hypoxemic population and is recommended in the setting of hypoxemic respiratory failure, post-extubation in selected patients, and in the postoperative setting for high-risk patients after cardiac or thoracic surgery [5, 9]. While the majority of evidence for HFNC is in the setting of acute hypoxemic respiratory failure, it is of increasing interest as an alternative to NIV in hypercapnic respiratory failure. Physiological studies suggest that the high gas flows of HFNC may improve ventilation by increasing mean airway pressure and washout of dead space, all while being more comfortable and tolerable by the patient [10–12]. Initial observational studies have demonstrated improvement in hypercapnia with the use of HFNC [13, 14].

Hence, our objective was to conduct a systematic review and meta-analysis to determine the efficacy and safety of HFNC compared to NIV for adults with acute hypercapnic respiratory failure. While previous systematic reviews have compared HFNC to NIV for the treatment of hypercapnia, they have important limitations, such as including heterogeneous patient populations [15, 16]. Additionally, these systematic reviews do not include several recently published randomized clinical trials (RCTs) [17, 18]. We hypothesized that there would be no increased risk of mortality when HFNC is used compared to NIV, but potentially an increased risk of intubation.

## Methods

### Study selection

We included parallel-group and crossover RCTs that enrolled adults ≥ 18 years old presenting with acute hypercapnic respiratory failure, defined as a pH < 7.35 or partial pressure of carbon dioxide (PaCO_2_) > 45 mmHg, regardless of the etiology. Eligible studies compared HFNC (any setting or duration) to NIV (defined as those with bi-level positive airway pressure, regardless of setting, interface or duration). Studies reporting on at least one of the following outcomes were included: the primary outcome of mortality at longest follow-up, or secondary outcomes of endotracheal intubation and invasive mechanical ventilation, hospital length of stay (LOS), Intensive Care Unit (ICU) LOS, change in PaCO_2_, change in partial pressure of oxygen (PaO_2_), respiratory rate (measured at the end of treatment), comfort (measured on a 10-point analog scale at the longest duration of treatment), or dyspnea (defined by the Borg scale taken at longest follow up). In addition to study inclusion criteria, collected characteristics were patient age, patient sex, Acute Physiologic Assessment and Chronic Health Evaluation II (APACHE II) score, and characteristics of the intervention and control group. We excluded pseudo- or quasi-randomized trials, and studies including patients with tracheostomy or were immediately post-extubation. Ethics approval was not obtained as no patient-level data was used in this systematic review.

### Electronic search strategy

We searched EMBASE, MEDLINE, and the Cochrane library from inception to October 2021 (Additional file 1: Tables S1 and S2), without limits on publication status or language. Existing systematic reviews and meta-analyses were cross-referenced for potentially eligible studies. Retrieved references were uploaded to Covidence for data management and screening (Covidence systematic review software, Veritas Health Innovation, Melbourne, Australia).

### Data collection and analysis

Two independent pairs of reviewers (SO, EH; and NO, KL) screened titles and abstracts in duplicate, and any potentially relevant study was advanced to full-text review. Full-text review was also performed in duplication, with disagreements resolved through discussion. Reviewers (NO and KL) extracted relevant data from eligible trials independently and in duplicate using a pre-designed and piloted data extraction form.

### Risk of bias

Two reviewers (NO and KL) independently assessed the studies for risk of bias (RoB) using the original Cochrane risk-of-bias tool (RoB) for randomized trials [19]. RoB was assessed in each study by outcome with reference to: random sequence generation, allocation concealment, blinding of participants and personnel, blinding of outcome assessors, incomplete outcome data, selective reporting, and other biases. RoB was judged to be low if all domains had low risk of bias. High risk of bias in any domain resulted in a high-risk categorization for that outcome. Disagreements were resolved by discussion between the two reviewers, or with arbitration with senior authors (KL and SO) if needed.

## Analysis

### Measurement of treatment effect

We uploaded extracted data into RevMan (Review Manager, version 5.3. Copenhagen: The Nordic Cochrane Centre, The Cochrane Collaboration, 2014) for meta-analysis. We used the DerSimonian and Laird random-effects model to pool the weighted effect of estimates across all studies [20]. The Mantel–Haenszel method was used to estimate study weights for dichotomous outcomes and inverse variance for continuous outcomes. Pooled relative risks (RRs), mean differences (MDs) or standardized mean differences (SMDs) were calculated for dichotomous and continuous outcomes (respectively), with corresponding 95% confidence intervals (CIs). When required, medians and interquartile ranges were converted to means and standard deviations for the purpose of the meta-analysis [21]. Funnel plots were inspected to assess for any publication bias if ten or more studies existed for that outcome [22].

### Unit of analysis

For all main outcomes, only one pair-wise comparison was conducted so the same groups of participants were only included once in the meta-analysis. For crossover trials, data was extracted only from the first phase to avoid the potential of carry-over effects.

### Heterogeneity and subgroup analysis

Statistical heterogeneity was assessed using Chi^2^ and *I*^2^ statistics. A Chi^2^ *P* value of < 0.1 or an *I*^2^ > 50% was pre-determined to meet the criteria of significant heterogeneity [23]. Significant heterogeneity between studies was explored through predefined subgroup analyses to investigate whether certain baseline factors influenced treatment effects. We had two planned subgroup analyses: etiology of hypercapnic respiratory failure (AECOPD vs non-AECOPD diagnoses, hypothesizing a larger treatment effect in AECOPD subgroup), and severity of acidosis (7.30–7.34 vs < 7.30, hypothesizing larger treatment effect in the 7.30–7.34 subgroup).

### Sensitivity analysis

We conducted a pre-specified sensitivity analysis restricted to studies without concerns for risk of bias. We hypothesized that the treatment effect would be smaller after excluding studies with some or high concerns of bias. Additionally, we conducted a post hoc analysis excluding one study (Wang et al*.*) which was only available as an abstract [15, 24].

### Assessing the certainty of evidence

Certainty of evidence for all major outcomes was assessed using the Grading of Recommendations Assessment, Development and Evaluation (GRADE) approach [25]. GRADE considers individual study risk of bias, inconsistency, indirectness, imprecision, and publication bias. This was performed by two reviewers (NO and KL) independently and in duplicate for each outcome. Certainty of evidence was ranked as very low, low, moderate, or high.

GRADEpro software [GRADEpro GDT: GRADEpro Guideline Development Tool (Software), McMaster University, 2020] was used to prepare the Summary of findings (SoF) table (Table 1) [26]. Justification of all decisions are presented in the footnotes. We used minimal important differences to assist in judgements of imprecision. The minimal important differences can be found in the SoF table footnotes and all values were based on clinical judgements post hoc.

**Table 1:** Summary of Findings

Certainty assessment							No of patients		Effect		Certainty	Importance
No of studies	Study design	Risk of bias	Inconsistency	Indirectness	Imprecision	Other considerations	HFNC	NIV	Relative (95% CI)	Absolute (95% CI)		
*Mortality*												
4	Randomized trials	Not serious^a^	Not serious	Not serious	Very serious^b^	None	18/127 (14.2%)	21/123 (17.1%)	RR 0.86 (0.48 to 1.56)	2 fewer per 100 (from 9 fewer to 10 more)	⨁⨁◯◯ Low	
*Intubation*												
4	Randomized trials	Not serious^a^	Not serious	Not serious	Very serious^c^	None	19/141 (13.5%)	23/134 (17.2%)	RR 0.80 (0.46 to 1.39)	3 fewer per 100 (from 9 fewer to 7 more)	⨁⨁◯◯ Low	
*ICU length of stay*												
2	Randomized trials	Not serious	Serious^d^	Not serious	Serious^e^	None	34	33	–	MD 0.08 higher (1.16 lower to 1.32 higher)	⨁⨁◯◯ Low	
*Hospital length of stay*												
4	Randomized trials	Not serious	Not serious	Not serious	Not serious^f^	None	178	174	–	MD 0.82 lower (1.83 lower to 0.2 higher)	⨁⨁⨁⨁ High	
*Comfort*												
2	Randomized trials	serious^g^	Serious^h^	Not serious	Serious^i^	None	49	52	–	SMD 0.32 lower (1.78 lower to 1.13 higher)	⨁◯◯◯ Very low	
*Dyspnea*												
4	Randomized trials	serious^g^	Not serious	Not serious	Serious^j^	None	98	93	–	MD 0.04 lower (0.54 lower to 0.45 higher)	⨁⨁◯◯ Low	
*PaO*_2_												
5	Randomized trials	Not serious^a^	Not serious	Not serious	Not serious^k^	None	215	212	–	MD 0.78 lower (4.18 lower to 2.62 higher)	⨁⨁⨁⨁ High	
*PaCO*_2_												
7	Randomized trials	Not serious^a^	Serious^l^	Not serious	Not serious^k^	None	245	242	–	MD 1.87 lower (5.34 lower to 1.6 higher)	⨁⨁⨁◯ Moderate	
*Respiratory rate*												
5	Randomized trials	Not serious^a^	Not serious	Not serious	Serious^m^	None	119	115	–	MD 0.85 lower (1.88 lower to 0.18 higher)	⨁⨁⨁◯ Moderate	

*CI* Confidence interval; *MD* Mean difference; *RR* Risk ratio; *SMD* Standardized mean difference

a. All probably low risk except for greater than 10% dropout rate and more outcomes reported than described in methods in single study

b. Minimally important difference threshold of 3% is crossed (likely wide CI due to small sample size) and very few events

c. Minimally important difference threshold of 5% is crossed (likely wide CI due to small sample size) and very few events

d. Point estimates significantly different, *I* squared > 50%

e. Minimally important difference threshold of 2 days.is met. Imprecision due to small sample size

f. Minimally important difference threshold of 2 days is met

g. Subjective outcome which will be significantly affected by lack of blinding

h. Significant heterogeneity, *I* squared > 50%, different point estimates without overlapping confidence intervals, *P* < 0.1

i. Minimally important difference threshold of 1SD was crossed. Imprecision due to small number of participants

j. Minimally important difference threshold of 1SD. Imprecision due to small number of participants

k. For the minimally important difference (threshold 15 mmHg), we considered no difference in PCO_2_ to be significant and there was a large number of total patients in the analysis

l. Significant difference in confidence intervals, borderline high *I* squared and *P* value < 0.1

m. Minimally important difference of 10 breaths per minute. Imprecision due to small number of participants

### Trial sequential analysis

We used trial sequential analysis (TSA) to determine if the required sample size to reach the threshold for statistical significance was met for the important outcomes of morality, intubation and ICU LOS. We performed these analyses using TSA software v. 0.9.5.10 Beta (Copenhagen Trial Unit, Center for Clinical Intervention Research, Rigshospitalet, Copenhagen, Denmark available at http://ctu.dk/tsa/). We constructed cumulative z-scores and the required information sizes (RIS) to definitively accept or refute the effect size of interest. We conducted primary TSA using an alpha of 0.05, power of 0.90 (beta 0.10), estimated diversity, unweighted control event proportions for binary outcomes and variances as estimated in the included trials for continuous outcomes. We defined relative risk reduction (RRR) of 15% as a clinically important difference for the outcomes of mortality and intubation and a mean difference (MD) of 24 h for the outcome of ICU LOS. Of note, the TSA was performed post hoc at the request of the journal.

## Results

### Screening

**Fig. 1:**
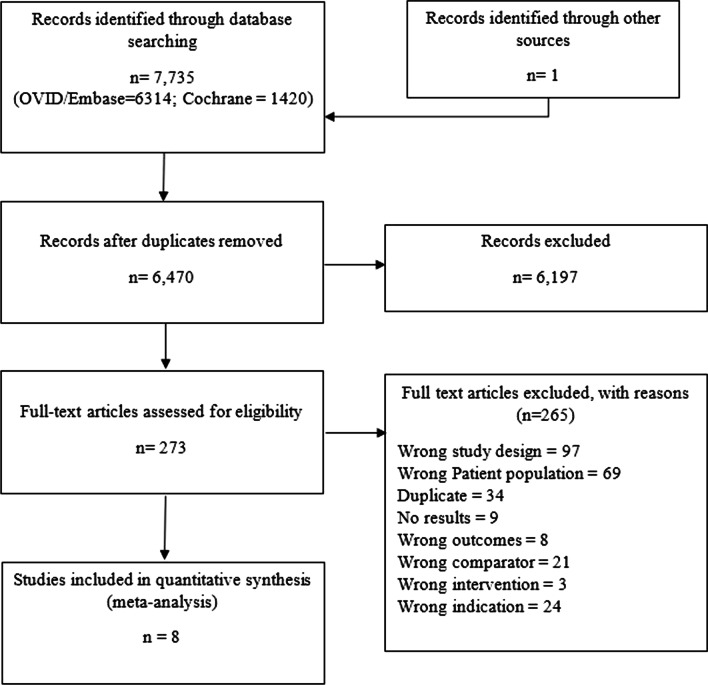
PRISMA flow diagram

Following the electronic search, 7735 studies were imported for screening and 4915 were screened by title and abstract after removal of duplicates (Fig. 1). Full-text review was completed for 273 studies and eight were included in the analysis [17, 18, 24, 27–31]. All studies except for one were published as full manuscripts [24]. Excluded studies and reasons for exclusion are available in the supplement (Additional file 1: Table S3).

### Characteristics of included studies

The eight studies included a total of 528 patients (Table 2) [17, 18, 24, 27–31]. The mean age of participants was 65.9 ± 11.8 years, with 43% being females. The mean APACHE II score was 21.0 ± 7.6. The mean pH of patients on presentation was 7.32 ± 0.04 and the mean PaCO_2_ was 64.33 ± 7.25 mmHg. All studies were limited to patients with acute hypercapnic respiratory failure. Six studies were parallel group RCTs [17, 18, 24, 27, 28, 32], and two were crossover trials [29, 31].‬

**Table 2:** Characteristics of included studies

Study	Study design	Patient characteristics	Setting	Inclusion criteria	Exclusion criteria by acidosis severity	Intervention	Control	Primary outcome
Cong et al. [28]	Parallel-group RCT	Mean age (SD), 67.52 (7.13) Female sex, 41.2% Mean APACHE II, NR Mean pH on presentation, 7.26 (NR) Mean PaCO_2_ on presentation, 72.51 mmHg	ICU	Patients with a diagnosis of AECOPD, admitted to ICU, receiving ventilation therapy	None	*N* = 84 HFNC at 30–35L/min 37 °C	*N* = 84 NIV by facemask. Settings: IPAP 10 cm H_2_O and EPAP 5 cm H_2_O, titrated by patient symptoms and oxygenation	Arterial blood gases at 12 h and 5 days after treatment
Cortegiani et al. [18]	Multicenter parallel-group RCT	Mean age, 75.5 (NR) Female sex, 19.5% Mean SAPS II 31.2 (NR) Mean pH on presentation, 7.30 Mean PaCO_2_ on presentation, 72.9 mmHg (NR)	Emergency department, ICU, or respiratory unit	Adults > 18 years old with a diagnosis of AECOPD, pH 7.25–7.35 with PaCO_2_ ≥ 55 mmHg	None	*N* = 40 HFNC initially set at 60L/min, 37 °C. Flows and temperature downregulated for tolerance	*N* = 39 NIV through full-face or oro-nasal mask Settings: pressure support ventilation, EPAP 3–5 cm H_2_O, titrated inspiratory pressure for tidal volume 6–8 mL/kg ideal body weight	Mean difference PaCO_2_ at 2 h post randomization
Doshi et al. [30]	Parallel-group RCT, predefined subgroup analysis	Median age, 62 (NR) Female sex, 52.3% Mean APACHE II, 30 (NR) Mean pH on presentation, 7.33 (NR) Mean PaCO_2_ on presentation, 60.3 mmHg (NR)	Emergency department	Adults > 18 presenting to the ED with acute respiratory failure, determined to need non-invasive positive pressure ventilation by physician assessment. Subgroup analysis of patients with a discharge diagnosis of AECOPD or acute hypercapnic respiratory failure. Impaired ventilation defined as elevated PaCO_2_ and pH < 7.35	None	*N* = 34 HFNC at 35L/min, temperature 35–37 °C and FiO_2_ 1.0. Adjustments made at discretion of treating physician	*N* = 31 NIV with an oronasal mask. Settings IPAP 10–20 cm H_2_O and EPAP 5–10 cm H_2_O with FiO_2_ 1.0. Adjustments made at discretion of treating physician	Change in PaCO_2_ and pH over time
Lee et al. [27]	Randomized controlled trial	Median age (IQR), 73 (66.5–79) Female sex, 43% Mean APACHE II, NR Mean pH on presentation (SD), 7.32 (0.03) Mean PaCO_2_ on presentation (SD), 54.5 (9.6)	Inpatients, location not specified	Adult patients ≥ 45 years with smoking history ≥ 10 pack years hospitalized with severe AECOPD. Moderate hypercapnic respiratory failure defined as requiring NIV after oxygen therapy of FiO_2_ > 50% for > 15 min and having a PaO_2_/FiO_2_ ratio of < 200 mmHg and PaCO_2_ > 45 mmHg with pH 7.25–7.35 on room air	None	*N* = 44 HFNC initiated at 35L/min titrated as tolerated to 45-60L/min. FiO_2_ initiated at > 50% and titrated for oxygen saturation of > 92%	*N* = 44 NIV delivered via nasal or full-face masks, based on patient comfort. Settings were in spontaneous/timed mode with initial IPAP at 10 cm H_2_O and EPAP 5 cm H_2_O, increased as tolerated over 1 h. Targeted tidal volumes of 7–10 mL/kg predicted body weight. FiO_2_ adjusted for oxygen saturation of > 92%	Intubation rate due to continuous hypoxia and hypercapnia
Papachatzakis et al. [17]	Parallel-group RCT	Mean age (SD), 77.0 (11.0) Female sex, 52.5% Mean APACHE (SD), 20.5 (7.6) Mean pH on presentation (SD), 7.4 (0.1) Mean PaCO_2_ on presentation, 61.2 mmHg (10.0)	Emergency department	Hypercapnic respiratory failure with PaCO_2_ > 45 mmHg	pH < 7.20	*N* = 20 HFNC initiated at 35L/min and titrated up as tolerated to 45-50L/min for SaO_2_ > 90% or per clinical order	*N* = 20 NIV, mask type not described. Settings: spontaneous/timed mode with pressures titrated by patient tolerance over 1 h for SaO_2_ > 90% or per clinical order	Not specified. All outcomes: intubation and mortality rate, length of hospitalization, duration of therapy, differences between vital signs, arterial blood gases, and comfort
Rezaei et al. [29]	Randomized crossover trial	Mean age, 61.27 (NR) Female sex, 20% Mean APACHE II, NR Mean pH on presentation, 7.32 (NR) Mean PaCO_2_ on presentation, 64.58 mmHg (NR)	Emergency department or ward	Patients between 18 and 65 years old with moderate to severe AECOPD and acute hypercapnic respiratory failure. Criteria for hypercapnia were pH between 7.25 and 7.35, PaCO_2_ > 45 mmHg	None	*N* = 15 HFNC initiated at flows of 15-35L/min at 37 °C for 30 min followed by a 1 h washout and switch to the alternate intervention Two patient groups in the study. The first started with HFNC and switched to NIV and the second started with NIV and switched to HFNC	*N* = 15 NIV delivered for 30 min followed by a 1 h washout and switch to the alternate intervention. Settings not described	Respiratory rate, heart rate, pH, dyspnea score, PaO_2_ and PaCO_2_
Sklar et al. [31]	Randomized crossover trial	Median age (IQR), 30 (23–34) Female sex, 8% Median APACHE II (IQR), 8 (7–9.5) Mean pH on presentation, NR Median transcutaneous CO_2_ on presentation (IQR), 53 (42–60)	Inpatients, location not specified	Adult patients > 18 years old with cystic fibrosis and clinical indication for NIV at the time of admission based on: clinical respiratory distress (respiratory rate > 24/min or accessory muscle use, PaCO_2_ > 45 mmHg from hospital admission, chronic nocturnal NIV now requiring daytime NIV, diurnal hypercapnia PaCO_2_ > 45 mmHg or transcutaneous CO_2_ > 40 mmHg in patients with serum bicarbonate ≥ 32 mmol/L	None	*N* = 15 30-min periods of time of HFNC at 55L/min if tolerated and FiO_2_ adjusted for oxygen saturation > 92% and temperature at 37 °C or 34 °C as per patient preference	*N* = N/A (crossover) 30 min periods of time of NIV with facemask. Settings adjusted by respiratory therapy team; details not specified. FiO_2_ adjusted for oxygen saturation of > 92%	Not specified All outcomes: oxygen saturation, transcutaneous CO_2_, respiratory rate, tidal volume, minute ventilation, diaphragm thickening fraction, dyspnea, comfort
Wang et al. [24]	Randomized controlled trial	Mean age, NR Female sex, NR Mean APACHE II, NR Mean pH on presentation, NR Mean PaCO_2_ on presentation, NR	NR	Patients with AECOPD, criteria not specified	Not specified	HFNC, settings not specified	NIV, settings not specified	Intervention failure (switch to alternate intervention or endotracheal intubation), endotracheal intubation, complications, 28 day survival

*NIV* Non-invasive ventilation; *HFNC* High-flow nasal cannula; *IPAP* Inspiratory positive airway pressure; *EPAP* Expiratory positive airway pressure; *PaCO*_2_ Partial pressure of carbon dioxide; *PaO*_2_ Partial pressure of oxygen; *FiO*_2_ Fraction of inspired oxygen; *APACHE II* Acute physiologic assessment and chronic health evaluation II score; *SAPS II* Simplified acute physiology score II; *NR* Not reported; *N/A* Not applicable

Five studies assessed the outcomes of HFNC vs. NIV in patients with AECOPD [18, 24, 27–29]. One study studied patients with cystic fibrosis [31] and two studies enrolled patients with any cause of hypercapnic respiratory failure [17, 32]. Two studies included patients in the emergency department (ED) [17, 30] and one limited to ICU patients [28]. Four studies had broad inclusion criteria of inpatients or admissions to the ED, ICU, or respiratory unit [18, 27, 29, 31]. Location of admission was not available for one study [24].

Inclusion criteria for pH and PaCO_2_ varied. Three studies set a limit of a pH ranging from 7.25 to 7.35 [18, 27, 29], whereas another required patients to have a pH > 7.20 [17]. One study’s inclusion criteria for hypercapnic respiratory acidosis was based on pH alone (< 7.35) and another was based on PaCO_2_ alone [31, 32]. Two studies did not set specific pH or CO_2_ cutoffs in their inclusion criteria [24, 28].

### Risk of bias

Risk of bias varied significantly based on the type of outcome measure (Additional file 1: Table S4). Risk was overall low for objective measures (mortality, intubation, hospital LOS, ICU LOS, respiratory rate, PaO_2_, and PaCO_2_) with the exception of one study which had a high loss to follow-up rate resulting in high risk of bias [27]. Two studies were deemed to be at potentially high risk of bias due to their funding [30, 31]. One study had high risk of bias due to selective reporting, with the addition of outcomes measured following trial registration [29]. Risk of bias was rated as high in all studies for the subjective outcomes of dyspnea and comfort in all studies due to lack of blinding.

## Outcomes

### Mortality

**Fig. 2:**
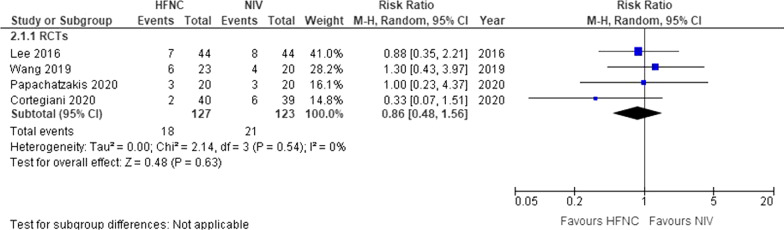
Mortality. *HFNC* High flow nasal cannula; *NIV* Non-invasive ventilation; *RCTs* Randomized controlled trials

Four studies (*n* = 250) reported on mortality at the longest follow-up [17, 18, 24, 27]. The use of HFNC compared to NIV did not demonstrate a difference (RR 0.86, 95% CI 0.48–1.56, *I*^2^ = 0%, low certainty) (Fig. 2). The absolute risk difference was − 2% (95% CI – 9–10) (Table 1).

### Endotracheal intubation

**Fig. 3:**
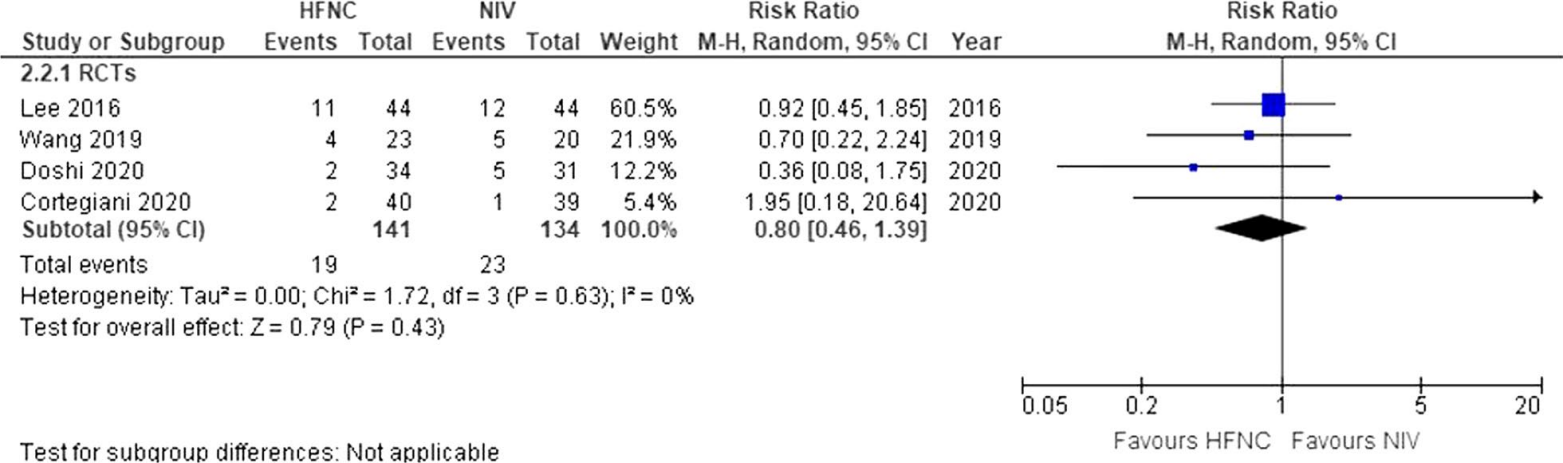
Intubation. *HFNC* High flow nasal cannula; *NIV* Non-invasive ventilation; *RCTs* Randomized controlled trials

Four studies (*n* = 275) reported on endotracheal intubation outcomes [18, 24, 27, 30]. The confidence interval was imprecise, indicating no difference in outcome (RR 0.80, 95% CI 0.46–1.39, *I*^2^ = 0%, low certainty) (Fig. 3). This translates into an absolute risk difference of − 3% (95% CI – 9–7) (Table 1).

### ICU length of stay

**Fig. 4:**
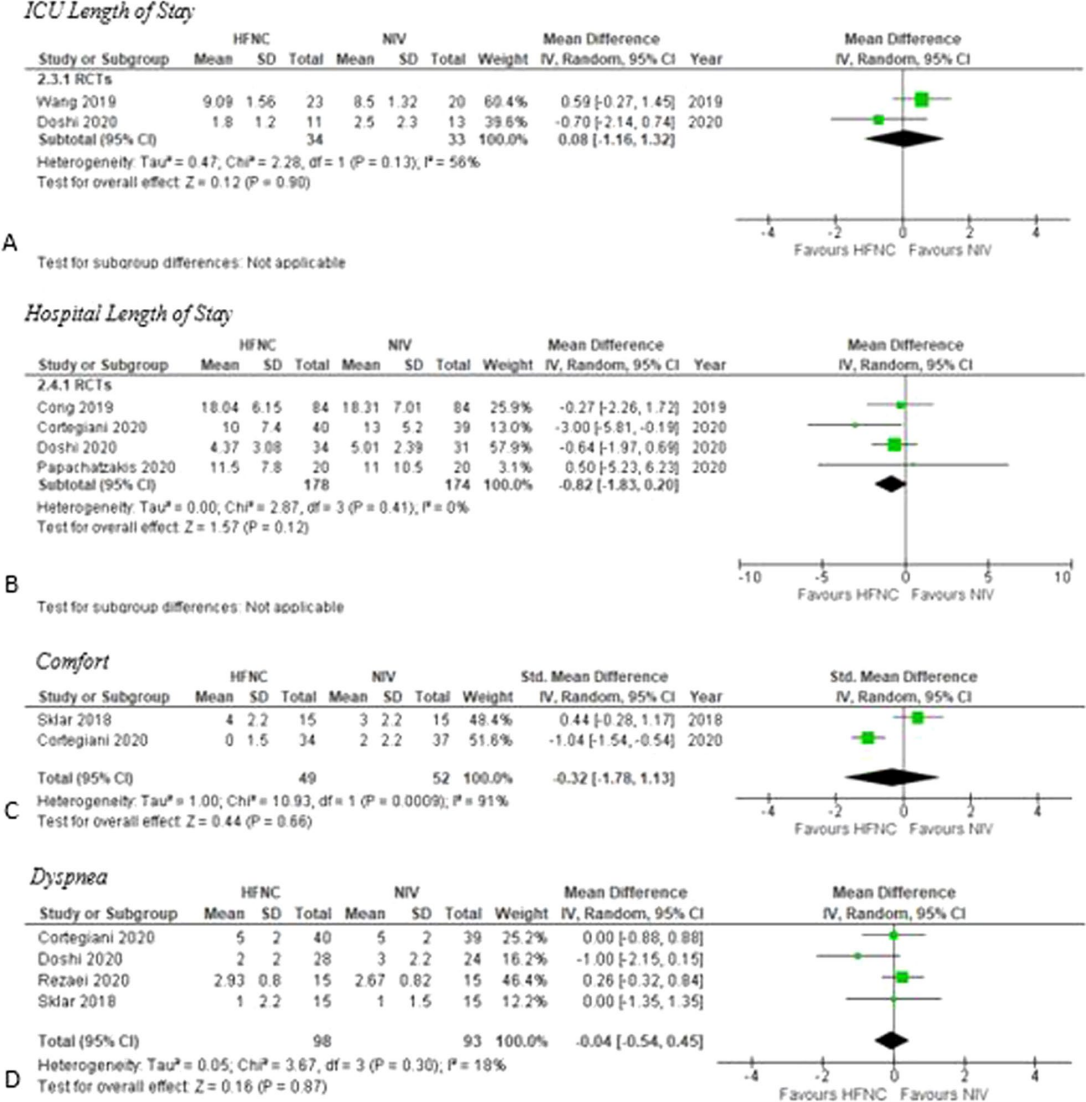
Secondary Outcomes. *HFNC* High flow nasal cannula; *NIV* Non-invasive ventilation; *RCTs* Randomized controlled trials

The pooled point estimate from two studies (*n* = 67) demonstrated no statistically significant reduction in duration of ICU LOS when HFNC was used compared to NIV (MD 0.08 days, 95% CI − 1.16–1.32, *I*^2^ = 56%, low certainty) (Fig. 4) [24, 30].

### Hospital length of stay

Four studies (*n* = 352) measured hospital LOS [17, 18, 28, 30]. HFNC did not change the duration of hospital LOS compared to NIV (MD − 0.82 days, 95% CI − 1.83–0.20, *I*^2^ = 0%, high certainty) (Fig. 4).

### Comfort

Two studies (*n* = 101) measured comfort at the longest duration of treatment [18, 31]. The comfort of patients on HFNC did not differ from those receiving NIV (SMD − 0.32 points, 95% CI − 1.78–1.13, *I*^2^ = 91%, very low certainty) (Fig. 4) [18, 31].

### Dyspnea

Four studies (*n* = 191) reported on dyspnea using a Borg scale or equivalent [33, 34]. The pooled estimate showed no clinically important difference in dyspnea scores after treatment when HFNC was used compared to NIV (MD − 0.04 points, 95% CI − 0.54–0.45, *I*^2^= 18%, very low certainty) (Fig. 4) [18, 29–31].

### Respiratory rate

Five studies (*n* = 234) reported on respiratory rate [17, 18, 29–31]. There was no statistical difference in the respiratory rate between the two interventions (MD − 0.85 breaths/min, 95% CI − 1.88–0.18, *I*^2^ = 0%, low certainty).

### PaO_2_ and PaCO_2_

Five studies (*n* = 427) measured change in PaO_2_, and no difference in PaO_2_ level was observed (MD − 0.78 mmHg, 95% CI − 4.18–2.62, *I*^2^ = 0%, high certainty) (Additional file 1: Fig. S1) [17, 18, 27, 28, 30].

Pooling the results across seven studies (*n* = 487) showed no difference in change in PaCO_2_ between those treated with HFNC versus NIV (MD − 1.87 mmHg, 95% CI − 5.34–1.60 mmHg, *I*^2^ = 47%, moderate certainty) (Additional file 1: Fig. S2) [17, 18, 27–31].

## Subgroup and sensitivity analyses

Subgroup analysis by AECOPD category for the comfort outcome demonstrated a subgroup effect favoring HFNC in AECOPD (P-interaction = 0.001, I2 = 90.6%; Additional file 1: Fig. S3), however this analysis only included two studies. There was no subgroup effect for the remaining outcomes (Additional file 1: Figs. S4–S6). We were unable to conduct subgroup analyses by severity of acidosis.

Sensitivity analyses excluding high risk of bias trials or excluding the only study published as an abstract [24] did not alter the results of analyzed outcomes (Additional file 1: Figs. S7–S16).

The TSA for all outcomes was inconclusive, as they did not meet the RIS and the boundaries for benefit, harm, or futility were not crossed (Additional file 1: Figs. S17–S19).

## Discussion

In this systematic review and meta-analysis of eight RCTs (*n* = 528 patients), there was no difference in the need for endotracheal intubation (low certainty), mortality at longest follow-up (low certainty), ICU LOS (low certainty), hospital LOS (high certainty), or change in PaCO_2_ (moderate certainty) or PaO_2_ (high certainty) when HFNC was compared to NIV in patients with hypercapnic respiratory failure.

While NIV use may reduce risks of death and endotracheal intubation in patients with hypercapnic respiratory failure compared to conventional oxygen therapy, it is not tolerated by all patients, leaving physicians with few options other than proceeding with endotracheal intubation. HFNC is increasingly used in acute hypoxic respiratory failure, but theoretically may also assist in ventilation, potentially with increased comfort and tolerance compared to NIV. Recent ERS guidelines made a conditional recommendation for a trial of NIV prior to use of HFNC in patients with COPD and acute hypercapnic respiratory failure, noting that there is high certainty that NIV reduces intubation, and that more evidence was needed before HFNC could be considered equivalent or superior to NIV. It was noted that there was limited evidence outside of COPD, and that more information was needed to identify patient populations where HFNC could be trialed prior to NIV.

Overall, our results are similar to those of previous systematic reviews, even accounting for the differences in trial selection [15, 16]. Specifically, previous systematic reviews included post-extubation studies. This population is excluded in the current analysis as they may have reasons other than hypercapnic respiratory failure for requiring reintubation, including post-extubation stridor, ineffective cough, and secretion management [35].

The study has a number of strengths, including use of a peer-reviewed electronic search strategy, with iterative searches up to October 2021. Screening, risk of bias, and certainty of evidence assessment were done in duplicate. We considered a priori subgroups of patient populations, hypothesizing that effect of HFNC may be different in patients with AECOPD.

The interpretation of these results is limited by the relatively small number of studies and patients, which resulted in imprecision of the results. As an emerging clinical entity, many studies evaluated physiologic variables rather than the patient-important outcomes of mortality and intubation. Additionally, patient goals of care (whether or not they would be candidates for intubation) were not reported and would be valuable for assessment of the mortality and intubation outcomes. Although a lack of significance may be seen as a limitation, this simply means that we have identified a knowledge gap and there needs to be a call to action by critical care researchers to expand on this important topic. This is further supported with the TSA. Some subgroup analyses may be underpowered due to small number of included studies. Moreover, we hypothesized that patients with more severe respiratory acidosis treated with HFNC may require intubations more frequently than those treated with NIV. Unfortunately, we were unable to complete an analysis based on degree of acidosis due to a complete lack of subgroup data. Study populations were also heterogenous, without consistent stratification between AECOPD and non-AECOPD causes of hypercapnic respiratory failure, thereby limiting conclusions on this specific question. Lastly, we were unable to examine funnel plots to detect publication bias given the small number of available studies. We attempted to minimize publication bias through extensive searches of databases, employing no language restrictions, and discussing the findings with experts in the field. Although, this systematic review protocol was not registered or published, this study was a sub-study of an ongoing clinical practice guideline that follows pre-specified methodology. As indicated above, the only post hoc analysis was a sensitivity analysis where we excluded abstracts. All other decisions were made a priori*.*

## Conclusions

In summary, emerging evidence is inconclusive in identifying whether HFNC may be an alternative to NIV for patients with hypercapnic respiratory failure. Further trials, such as an upcoming randomized non-inferiority trial [36], may improve the precision of the estimates.

## Supplementary Information

**Additional file 1: Table S1.** Embase and Medline Search Results. **Table S2.** Cochrane Central Search Results. **Table S3.** Excluded Studies. **Table S4.** Risk of Bias Table. **Fig. S1.** Forest plot of mortality—subgroup analysis by risk of bias. **Fig. S2.** Forest plot of mortality—subgroup analysis excluding Wang et al. **Fig. S3.** Forest plot of intubation—subgroup analysis by risk of bias. **Fig. S4.** Forest plot of intubation—subgroup analysis excluding Wang et al. **Fig. S5.** Forest plot of ICU Length of Stay—subgroup analysis by risk of bias. **Fig. S6.** Forest plot of ICU Length of Stay—subgroup analysis excluding Wang et al. **Fig. S7.** Forest plot of Hospital Length of Stay—subgroup analysis by risk of bias. **Fig. S8.** Forest plot of change in comfort—subgroup analysis by AECOPD studies alone. **Fig. S9.** Forest plot of change in dyspnea—subgroup analysis by AECOPD studies alone. **Fig. S10.** Forest plot of change in respiratory rate—subgroup analysis by AECOPD studies alone. **Fig. S11.** Forest plot of respiratory rate—subgroup analysis by risk of bias. **Fig. S12.** Forest plot of change in PO_2_. **Fig. S13.** Forest plot of change in PO_2_—subgroup analysis by risk of bias. **Fig. S14.** Forest plot of change in PCO_2_. **Fig. S15.** Forest plot of change in PCO_2_—subgroup analysis by AECOPD studies alone. **Fig. S16.** Forest plot of change in PCO_2_—subgroup analysis by risk of bias. **Fig. S17.** Trial sequential analysis for mortality. **Fig. S18.** Trial sequential analysis for intubation. **Fig. S19.** Trial sequential analysis for ICU length of stay.

## Data Availability

The datasets used and/or analyzed during the current study are available from the corresponding author on reasonable request.

## Abbreviations

AECOPD: Acute exacerbation of chronic obstructive pulmonary disease
APACHE: Acute physiologic assessment and chronic health evaluation
ARR: Absolute risk reduction
BIPAP: Bi-level positive airway pressure
CO_2_: Carbon dioxide
CI: Confidence interval
ED: Emergency department
EPAP: Expiratory positive airway pressure
FiO_2_: Fraction of inspired oxygen
GRADE: Grading of recommendations assessment, development and evaluation
HFNC: High-flow nasal cannula
ICU: Intensive care unit
IPAP: Inspiratory positive airway pressure
LOS: Length of stay
MD: Mean difference
NIV: Non-invasive ventilation
PaCO_2_: Partial pressure of carbon dioxide
PaO_2_: Partial pressure of oxygen
PICO: Population, intervention, comparator, outcome
RR: Relative risk
RCT: Randomized controlled trial
SMD: Standardized mean difference
ROB: Risk of bias
RIS: Required information size
RRR: Relative risk reduction
SoF: Summary of findings
TSA: Trial sequential analysis
