# Supplementary material for: High-flow nasal cannula versus non-invasive ventilation for acute hypercapnic respiratory failure in adults: a systematic review and meta-analysis of randomized trials
Source: Crit Care. 2022 Nov 9;26:348. doi: 10.1186/s13054-022-04218-3 (PMC9648030; doi:10.1186/s13054-022-04218-3)
Supplement: Supplementary file 1 — Additional file 1: Table S1. Embase and Medline Search Results. Table S2. Cochrane Central Search Results. Table S3. Excluded Studies. Table S4. Risk of Bias Table. Fig. S1. Forest plot of mortality—subgroup analysis by risk of bias. Fig. S2. Forest plot of mortality—subgroup analysis excluding Wang et al. Fig. S3. Forest plot of intubation—subgroup analysis by risk of bias. Fig. S4. Forest plot of intubation—subgroup analysis excluding Wang et al. Fig. S5. Forest plot of ICU Length of Stay—subgroup analysis by risk of bias. Fig. S6. Forest plot of ICU Length of Stay—subgroup analysis excluding Wang et al. Fig. S7. Forest plot of Hospital Length of Stay—subgroup analysis by risk of bias. Fig. S8. Forest plot of change in comfort—subgroup analysis by AECOPD studies alone. Fig. S9. Forest plot of change in dyspnea—subgroup analysis by AECOPD studies alone. Fig. S10. Forest plot of change in respiratory rate—subgroup analysis by AECOPD studies alone. Fig. S11. Forest plot of respiratory rate—subgroup analysis by risk of bias. Fig. S12. Forest plot of change in PO2. Fig. S13. Forest plot of change in PO2—subgroup analysis by risk of bias. Fig. S14. Forest plot of change in PCO2. Fig. S15. Forest plot of change in PCO2—subgroup analysis by AECOPD studies alone. Fig. S16. Forest plot of change in PCO2—subgroup analysis by risk of bias. Fig. S17. Trial sequential analysis for mortality. Fig. S18. Trial sequential analysis for intubation. Fig. S19. Trial sequential analysis for ICU length of stay. [file 13054_2022_4218_MOESM1_ESM.docx]

**Supplementary Materials: e-Appendix 1**

**Table of Contents**

**e-Table 1:** Embase and Medline Search Results

**e-Table 2:** Cochrane Central Search Results

**e-Table 3:**  Excluded Studies

**e-Table 4:**  Risk of Bias Table

**e-Figure 1:** Forest plot of change in PaO_2_

**e-Figure 2:** Forest plot change in PaCO_2_

**e-Figure 3**: Forest plot of change in comfort - subgroup analysis by AECOPD studies alone

**e-Figure 4:** Forest plot of change in dyspnea - subgroup analysis by AECOPD studies alone

**e-Figure 5**: Forest plot of change in respiratory rate - subgroup analysis by AECOPD studies alone

**e-Figure 6:** Forest plot of change in PaCO_2_ - subgroup analysis by AECOPD studies alone

**e-Figure 7**: Forest plot of mortality - subgroup analysis by risk of bias

**e-Figure 8:** Forest plot of intubation - subgroup analysis by risk of bias

**e-Figure 9**: Forest plot of ICU Length of Stay - subgroup analysis by risk of bias

**e-Figure 10:** Forest plot of Hospital Length of Stay - subgroup analysis by risk of bias

**e-Figure 11:** Forest plot of respiratory rate - subgroup analysis by risk of bias

**e-Figure 12:** Forest plot of change in PaO_2_ - subgroup analysis by risk of bias

**e-Figure 13:** Forest plot of change in PaCO_2_ - subgroup analysis by risk of bias

**e- Figure 14**: Forest plot of mortality - subgroup analysis excluding Wang et al.

**e-Figure 15**: Forest plot of intubation - subgroup analysis excluding Wang et al.

**e-Figure 16**: Forest plot of ICU Length of Stay - subgroup analysis excluding Wang et al.

**e-Figure 17:** Trial sequential analysis for mortality

**e-Figure 18:** Trial sequential analysis for intubation

**e-Figure 19:** Trial sequential analysis for ICU length of stay

**e-Table 1:** Embase and Medline Search Results (April 16 search, updates were completed on August 4 2020, January 6, and October 5 2021)

**Concept #1: High-Flow Nasal Cannula**

Oxygen Inhalation Therapy/ use ppez

Oxygen Therapy/ use oemezd

(high-flow* OR highflow* OR high velocit* OR highvelocit*).mp.

(cannula?).mp.

exp Nasal Cannula/ use oemezd

*Nasal Cannula/ use oemezd

((high-flow* OR highflow* OR high velocit* OR highvelocit*) ADJ3 (nasal cannula* OR nasal insufflation* OR nasal oxygen* OR nasal therap* OR oxygen therap*)).mp,dv.

((high-flow* OR highflow* OR high velocit* OR highvelocit*) ADJ (cannula? OR insufflation* OR nasal OR oxygen* OR therap*)).mp,dv.

(HFNC OR HHFNC OR HFNO OR HHFN OR Airvo* OR Optiflow).mp,dv.

((trans-nasal* OR transnasal*) ADJ humidified rapid insufflation ventilatory exchang*).mp,dv.

*Ovid*

Database(s): **Embase**1974 to 2020 April 14, OVID Medline Epub Ahead of Print, In-Process & Other Non-Indexed Citations, Ovid MEDLINE(R) Daily and Ovid MEDLINE(R) 1946 to Present
Search Strategy:

| **#** | **Searches** | **Results** |
| --- | --- | --- |
| 1 | Oxygen Inhalation Therapy/ use ppez | 14203 |
| 2 | Oxygen Therapy/ use oemezd | 28628 |
| 3 | (high-flow* or highflow* or high velocit* or highvelocit*).mp. | 27883 |
| 4 | cannula?.mp. | 57199 |
| 5 | (1 or 2) and 3 and 4 | 1257 |
| 6 | exp Nasal Cannula/ use oemezd | 4103 |
| 7 | 3 and 6 | 1814 |
| 8 | *Nasal Cannula/ use oemezd | 907 |
| 9 | ((high-flow* or highflow* or high velocit* or highvelocit*) adj3 (nasal cannula* or nasal insufflation* or nasal oxygen* or nasal therap* or oxygen therap*)).mp,dv. | 3750 |
| 10 | ((high-flow* or highflow* or high velocit* or highvelocit*) adj (cannula? or insufflation* or nasal or oxygen* or therap*)).mp,dv. | 4800 |
| 11 | (HFNC or HHFNC or HFNO or HHFN or Airvo* or Optiflow).mp,dv. | 1912 |
| 12 | ((trans-nasal* or transnasal*) adj humidified rapid insufflation ventilatory exchang*).mp,dv. | 136 |
| 13 | or/5,7-12 | 5336 |
| 14 | remove duplicates from 13 | 3756 |

**e-Table 2:** Cochrane Library Search Results (April 16 search, updates were completed on August 4 2020, January 6, and October 5 2021)

**The Cochrane Library (CENTRAL)**

ID Search Hits

#1 MeSH descriptor: [Oxygen Inhalation Therapy] this term only 1116

#2 (high-flow* OR highflow* OR high velocit* OR highvelocit*):ti,ab,kw AND (cannula?):ti,ab,kw 774

#3 #1 AND #2 80

#4 (("high-flow" OR highflow OR "high velocitity") NEAR/3 (nasal cannula* OR nasal insufflation* OR nasal oxygen* OR nasal therap* OR oxygen therap*)):ti,ab,kw OR (("high-flow" OR highflow OR "high velocitity") NEXT (cannula? OR insufflation* OR nasal OR oxygen* OR therap*)):ti,ab,kw OR (HFNC OR HHFNC OR HFNO OR HHFN OR Airvo OR Optiflow):ti,ab,kw OR (("trans-nasal" OR transnasal) NEXT "humidified rapid insufflation ventilatory exchange"):ti,ab,kw in Trials 873

#5 #3 OR #4 with Publication Year from 2006 to 2020, in Trials 612

**e-Table 3:** Excluded Studies

| **Study number** | **Publication Year** | **First Author** | **Title** | **Exclusion Reason** | **Notes** |
| --- | --- | --- | --- | --- | --- |
| 3282 | 2019 | Adi, O. | Preliminary report: A randomized controlled trial comparing helmet continuous positive airway pressure (CPAP) vs high flow nasal cannula (HFNC) for treatment of acute cardiogenic pulmonary oedema in the emergency department | Wrong indication | CPAP vs HFNC for CHF |
| 3261 | 2019 | Sun | High flow nasal cannula oxygen therapy versus non-invasive ventilation for chronic obstructive pulmonary disease with acute-moderate hypercapnic respiratory failure: an observational cohort study [Corrigendum] | Wrong study design | Retrospective observational |
| 3653 | 2020 | Thille | Effect of postextubation high-flow nasal oxygen with noninvasive ventilation vs high-flow nasal oxygen alone on reintubation among patients at high risk of extubation failure: a randomized clinical trial | Wrong indication | Post extubation |
| 1700 | 2016 | Ansari | A Randomized Controlled Trial of High-Flow Nasal Oxygen (Optiflow) as Part of an Enhanced Recovery Program After Lung Resection Surgery | Wrong indication | Post lung resection |
| 2271 | 2017 | Arman | Effects of direct extubation to high-flow nasal cannula compared to standard nasal cannula in patients in the intensive care unit | Wrong indication | HFNC vs COT for post-extubation in hypoxic resp failure |
| 3616 | 2019 | Artaud-Macari | High-flow oxygen therapy vs non-invasive ventilation a prospective cross-over physiological study of alveolar recruitment in acute respiratory failure | Wrong indication | HFNC vs NIV for hypoxic resp failure, outcome - recruitment/lung volumes |
| 192 | 2010 | Austin | Effect of high flow oxygen on mortality in chronic obstructive pulmonary disease patients in prehospital setting: randomised controlled trial | Wrong comparator | HFNC vs COT for COPDE in prehospital setting |
| 338 | 2012 | Austin | Effect of high flow oxygen on mortality in chronic obstructive pulmonary disease patients in prehospital setting: Randomized controlled trial | Duplicate | Duplicate (#192) |
| 1346 | 2015 | Azevedo | High flow nasal cannula oxygen (HFNC) versus non-invasive positive pressure ventilation (NIPPV) in acute hypoxemic respiratory failure. A pilot randomized controlled trial | Wrong indication | HFNC vs NIV for hypoxic respiratory failure |
| 2580 | 2018 | Azoulay | Effect of High-Flow Nasal Oxygen vs Standard Oxygen on 28-Day Mortality in Immunocompromised Patients With Acute Respiratory Failure: The HIGH Randomized Clinical Trial | Wrong comparator | HFNC vs COT for hypoxic respiratory failure |
| 3649 | 2020 | Bae | High-Flow Nasal Cannula Oxygen Therapy Can Be Effective for Patients in Acute Hypoxemic Respiratory Failure with Hypercapnia: a Retrospective, Propensity Score-Matched Cohort Study | Wrong study design | HFNC for hypercapnic vs not hypercapnic in hypoxic resp failure, retrospective cohort |
| 1108 | 2015 | Bell | Randomised control trial of humidified high flow nasal cannulae versus standard oxygen in the emergency department | Wrong comparator | HFNC vs COT in pts with dyspnea in ED |
| 2236 | 2017 | Blaudzun | A randomised controlled trial of high flow nasal oxygen (OptiflowTM) in high risk cardiac surgical patients | Wrong indication | HFNC vs COT for postop cardiac surgery pts |
| 2230 | 2017 | Brainard | Heated humidified high-flow nasal cannula oxygen after thoracic surgery - A randomized prospective clinical pilot trial | Wrong indication | HFNC vs COT post thoracic surgery |
| 2135 | 2016 | Brainard | Heated high-flow nasal cannula oxygen after thoracic surgery: A pilot randomized controlled trial | Duplicate | Duplicate (#2230) |
| 3994 | 2020 | Cho | Comparison of Post-extubation Outcomes Associated with High-Flow Nasal Cannula vs. Conventional Oxygen Therapy in Patients at High Risk of Reintubation: a Randomized Clinical Trial | Wrong comparator | HFNC vs COT for post-extubation (prevent reintubation) |
| 1088 | 2015 | Corley | Direct extubation onto high-flow nasal cannulae post-cardiac surgery versus standard treatment in patients with a BMI >=30: a randomised controlled trial | Wrong comparator | HFNC vs COT in obese pts post cardiac surgery |
| 560 | 2011 | Cuquemelle | Heated and humidified high flow oxygen therapy reduces discomfort during hypoxemic respiratory failure | Wrong comparator | HFNC vs COT in hypoxemic respiratory failure |
| 319 | 2012 | Cuquemelle | Heated and humidified high-flow oxygen therapy reduces discomfort during hypoxemic respiratory failure | Duplicate | Duplicate (#560) |
| 2175 | 2017 | Delorme | Effects of High-Flow Nasal Cannula on the Work of Breathing in Patients Recovering From Acute Respiratory Failure | Wrong study type | HFNC at increasing flow rates for symptom relief in hypoxic and hypercapnic resp failure, non-randomized observational |
| 3980 | 2020 | Devraj | Effect on oxygenation with high flow nasal cannula (HFNC) vs conventional oxygen therapy (Venturi mask) after extubation in patients undergoing cardiac surgery | Wrong comparator | HFNC vs Venturi mask in cardiac surgery pts post extubation |
| 2774 | 2018 | DiMussi | Physiological evaluation of high flow oxygen therapy versus standard oxygen therapy effects in tracheostomized patients | Wrong population | HFNC vs COT for WOB in tracheostomized pts |
| 3489 | 2019 | DoCampo | Implementation of HFNO and reduction in the number of AECOPD needing NIV in a rural hospital | Wrong study type | Retrospective observational |
| 2768 | 2018 | Doshi | High-Velocity Nasal Insufflation in the Treatment of Respiratory Failure: A Randomized Clinical Trial | Wrong population | HFNC vs NIV for all respiratory failure |
| 4399 | 2020 | Fakharian | Comparison of high-flow oxygenation to noninvasive ventilation in COPD exacerbation | Duplicate | Duplicate (#4625) |
| 1067 | 2015 | Fernandez | High-flow oxygen therapy for extubation failure prevention in high-risk critically ill patients: A randomized multicenter trial | Wrong comparator | HFNC vs COT for post-extubation resp failure |
| 2147 | 2017 | Fernandez | High-flow nasal cannula to prevent postextubation respiratory failure in high-risk non-hypercapnic patients: a randomized multicenter trial | Duplicate | Duplicate (#1067) |
| 3224 | 2019 | Ferrando | High-flow nasal cannula oxygenation reduces postoperative hypoxemia in morbidly obese patients: a randomized controlled trial | Wrong comparator | HFNC vs COT post- extubation in bariatric pts |
| 1301 | 2016 | Frat | High-flow oxygen therapy through a nasal cannula in immunocompromised patients with acute hypoxemic respiratory failure | Wrong population | HFNC vs COT vs NIV for hypoxic resp failure immunocompromised |
| 1300 | 2016 | Frat | Effect of non-invasive oxygenation strategies in immunocompromised patients with severe acute respiratory failure: a post-hoc analysis of a randomised trial | Duplicate | Duplicate (#1301) |
| 1062 | 2015 | Frat | High-flow oxygen through nasal cannula in acute hypoxemic respiratory failure | Wrong population | HFNC vs COT vs NIV for hypoxic resp failure |
| 1299 | 2016 | Futier | Effect of early postextubation high-flow nasal cannula vs conventional oxygen therapy on hypoxaemia in patients after major abdominal surgery: a French multicentre randomised controlled trial (OPERA) | Wrong comparator | HFNC vs COT for hypoxic resp failure post-extubation |
| 3203 | 2019 | Gandino | Conventional oxygen-therapy vs HFNC in hypercapnic ARF: Rates of upgrade to NIV | Wrong comparator | HFNC vs COT for hypercapnic respiratory failure |
| 3460 | 2020 | Geng | High-Flow Nasal Cannula: A Promising Oxygen Therapy for Patients with Severe Bronchial Asthma Complicated with Respiratory Failure | Wrong comparator | HFNC vs COT in severe asthma |
| 3449 | 2020 | Grieco | Physiological Comparison of High-Flow Nasal Cannula and Helmet Noninvasive Ventilation in Acute Hypoxemic Respiratory Failure | Wrong indication | HFNC vs Helmet NIV for hypoxic respiratory failure |
| 477 | 2013 | Grieco | Comfort during high-flow oxygen therapy through nasal cannula in critically ill patients: Effect of gas temperature and flow | Wrong study type | Observational study - HFNC in increasing flows for comfort in patients requiring oxygen |
| 2428 | 2018 | Guoqiang | Comparison of high flow nasal cannula with noninvasive ventilation in facilitating weaning COPD from invasive ventilation: A prospective randomized controlled study | Wrong population | HFNC vs NIV for post-extubation mgmt in AECOPD |
| 1267 | 2016 | Hernandez | Effect of Postextubation High-Flow Nasal Cannula vs Noninvasive Ventilation on Reintubation and Postextubation Respiratory Failure in High-Risk Patients: A Randomized Clinical Trial | Wrong population | HFNC vs NIV post-extubation in high-risk patients |
| 1042 | 2015 | Hernandez | High flow conditioned oxygen therapy for prevention of reintubation in critically ill patiets at high risk for extubation failure: A multicenter randomised controlled trial | Duplicate | Duplicate (#1257) |
| 1266 | 2016 | Hernandez | Effect of Postextubation High-Flow Nasal Cannula vs Conventional Oxygen Therapy on Reintubation in Low-Risk Patients: A Randomized Clinical Trial | Wrong population | HFNC vs COT post-extubation in low risk patients |
| 3434 | 2020 | Hu | Effect of high-flow nasal oxygen vs. Conventional oxygen therapy on extubation outcomes and physiologic changes for patients with high risk of extubation failure in the medical ICU: A tertiary center, randomized, controlled trial | Wrong population | HFNC vs COT post-extubation in high risk |
| 468 | 2013 | Hui | High-flow oxygen and bilevel positive airway pressure for persistent dyspnea in patients with advanced cancer: a phase II randomized trial | Wrong population | HFNC vs NIV for palliation of dyspnea |
| 4816 | 2020 | Fakharian | Comparison of the Efficacy of High-Flow Nasal Oxygen Therapy and Noninvasive Ventilation in patients with COPD Exacerbation | Duplicate | Duplicate (#4625) |
| 3124 | 2019 | Ischaki | Nasal high flow versus non-invasive ventilation in patients with acute exacerbation of chronic obstructive pulmonary disease | Results not published | HFNC vs NIV in hypercapnic resp failure - still recruiting |
| 3106 | 2019 | Jing | Comparison of high flow nasal cannula with noninvasive ventilation in chronic obstructive pulmonary disease patients with hypercapnia in preventing postextubation respiratory failure: A pilot randomized controlled trial | Wrong population | HFNC vs NIV for postextubation in hypercapnic patients |
| 1240 | 2016 | Jones | Randomized Controlled Trial of Humidified High-Flow Nasal Oxygen for Acute Respiratory Distress in the Emergency Department: The HOT-ER Study | Wrong indication | HFNC vs COT for hypoxic respiratory failure |
| 1241 | 2016 | Jones | Randomized Controlled Trial of Humidified High-Flow Nasal Oxygen for Acute Respiratory Distress in the Emergency Department: The HOT-ER Study | Duplicate | Duplicate (#1240) |
| 171 | 2010 | Kernick | HiNoon – high flow nasal oxygen or nonrebreather: a pilot randomised crossover trial | Wrong Comparator | HFNC vs COT for hypoxic respiratory failure (CO2 reported) |
| 2363 | 2018 | Kim | Effectiveness of high-flow nasal cannula oxygen therapy for acute respiratory failure with hypercapnia | Wrong study type | Retrospective study - HFNC for hypercapnia |
| 3922 | 2020 | Ko | Benefits of High-Flow Nasal Cannula Therapy for Acute Pulmonary Edema in Patients with Heart Failure in the Emergency Department: A Prospective Multi-Center Randomized Controlled Trial | Wrong indication | HFNC vs COT for pulmonary edema |
| 3004 | 2019 | Lee | Reduction of PaCO2 by high-flow nasal cannula in acute hypercapnic respiratory failure patients receiving conventional oxygen therapy | Wrong study type | Retrospective observational HFNC for hypercapnic vs non hypercapnic patients |
| 1195 | 2016 | Lee | The efficacy of high-flow nasal cannulae oxygen therapy in severe acute exacerbation of chronic obstructive pulmonary disease: A randomized controlled trial | Duplicate | Duplicate (#2324) |
| 1009 | 2015 | Lemiale | The effects of a 2-h trial of high-flow oxygen by nasal cannula versus Venturi mask in immunocompromised patients with hypoxemic acute respiratory failure: a multicenter randomized trial | Wrong indication | HFNC vs VM for hypoxic resp failure |
| 1556 | 2017 | Lemiale | High-Flow Nasal Cannula Oxygenation in Immunocompromised Patients With Acute Hypoxemic Respiratory Failure: A Groupe de Recherche Respiratoire en Reanimation Onco-Hematologique Study | Wrong indication | HFNC vs VM for hypoxic resp failure (subgroup analysis) |
| 1193 | 2016 | Lemiale | High-flow nasal cannula for acute respiratory failure in immunocompromised patients | Duplicate | Duplicate (#1556) |
| 2978 | 2019 | Liu | High-Flow Oxygen Therapy to Speed Weaning From Mechanical Ventilation: A Prospective Randomized Study | Wrong population | HFNC for pts on IMV for weaning |
| 2976 | 2019 | Longhini | High-Flow Oxygen Therapy After Noninvasive Ventilation Interruption in Patients Recovering From Hypercapnic Acute Respiratory Failure: A Physiological Crossover Trial | Wrong population | HFNC for patients post 24h NIV, recovering from acute hypercapnic resp failure |
| 1575 | 2017 | Longhini | High-flow oxygen therapy in hypercapnic patients recovering from an episode of acute on-chronic respiratory failure | Duplicate | Duplicate (#2976) |
| 714 | 2014 | Maggiore | Nasal high-flow versus Venturi mask oxygen therapy after extubation. Effects on oxygenation, comfort, and clinical outcome | Wrong population | HFNC vs VM post extubation |
| 1534 | 2017 | Makdee | High-Flow Nasal Cannula Versus Conventional Oxygen Therapy in Emergency Department Patients With Cardiogenic Pulmonary Edema: A Randomized Controlled Trial | Wrong indication | HFNC vs COT for pulmonary edema |
| 1533 | 2017 | Makdee | Efficacy of nasal high flow in emergency patients with cardiogenic pulmonary edema: A randomized controlled trial | Duplicate | Duplicate (#1534) |
| 3386 | 2020 | Matsuda | High-Flow Nasal Cannula May Not Reduce the Re-Intubation Rate after Extubation in Respiratory Failure Compared With a Large-Volume Nebulization-Based Humidifier | Wrong population | HFNC vs COT post extubation |
| 1522 | 2017 | Mauri | Optimum support by high-flow nasal cannula in acute hypoxemic respiratory failure: effects of increasing flow rates | Wrong indication | HFNC at different flow rates for hypoxemic respiratory failure |
| 2291 | 2018 | Mauri | Impact of flow and temperature on patient comfort during respiratory support by high-flow nasal cannula | Wrong indication | HFNC at different flow rates for hypoxemic respiratory failure effect on comfort |
| 2290 | 2018 | Mauri | Effects of temperature and flow on patients' comfort during respiratory support by high flow nasal cannula | Duplicate | Duplicate (#2291) |
| 1519 | 2017 | Mauri | Physiologic Effects of High-Flow Nasal Cannula in Acute Hypoxemic Respiratory Failure | Wrong indication | HFNC at different flow rates for hypoxemic respiratory failure |
| 2287 | 2018 | McKinstry | RCT of NHF therapy compared with NIV in hypercapnic COPD | Wrong population | HFNC vs NIV in stable hypercapnia |
| 3735 | 2019 | Mendil | The effect of high flow nasal oxygen in hematological malignancy patients with acute hypoxemic respiratory insufficiency: Prospective, single center, randomized controlled trial | Wrong indication | HFNC vs COT for hypoxemic resp failure in hematological malignancy patients |
| 2934 | 2019 | Menga | Helmet non-invasive ventilation versus high flow nasal oxygen in acute hypoxemic respiratory failure: Physiological effects | Wrong indication | HFNC vs helmet NIV for hypoxemic resp failure |
| 2890 | 2019 | Norkiene | Effectiveness of high-flow nasal oxygen therapy in management of acute hypoxemic and hypercapnic respiratory failure | Wrong study type | Retrospective study HFNC for hypercapnic resp failure vs hypoxemic |
| 2877 | 2019 | Pandya | Tolerance and safety of humidified high-flow nasal cannula oxygen therapy in patients hospitalized with an acute exacerbation of chronic obstructive pulmonary disease (COPD) | Wrong study type | Prospective observational HFNC for hypercapnic resp failure |
| 1876 | 2017 | Papachatzakis | High flow nasal cannula with warm humidified air versus non-invasive mechanical ventilation in respiratory failure type II | Wrong study type | Observational, no randomization described HFNC vs NIV for hypercapnia |
| 222 | 2011 | Parke | A preliminary randomized controlled trial to assess effectiveness of nasal high-flow oxygen in intensive care patients | Wrong indication | HFNC vs NIV for hypoxemic respiratory failure |
| 402 | 2013 | Parke | Open-label, phase II study of routine high-flow nasal oxygen therapy in cardiac surgical patients | Wrong population | HFNC vs NIV post extubation in cardiac surgery patients |
| 625 | 2012 | Parke | Randomised controlled trial of prophylactic nasal high flow oxygen after cardiac surgery | Duplicate | Duplicate (#402) |
| 2867 | 2019 | Pennisi | Early nasal high-flow versus Venturi mask oxygen therapy after lung resection: a randomized trial | Wrong population | HFNC vs VM post extubation after lung resection |
| 682 | 2014 | Perbet | High-flow nasal oxygen cannula versus conventional oxygen therapy to prevent postextubation lung aeration loss: A multicentric randomized control lung ultrasound study | Wrong population | HFNC vs COT post extubation |
| 1855 | 2017 | Pietzsch | Resource utilization and costs of high velocity nasal insufflation compared to non-invasive positive pressure ventilation for respiratory failure | Wrong outcome | Cost and resource utilization of HFNC |
| 1853 | 2017 | Pilcher | Physiological effects of titrated oxygen via nasal high-flow cannulae in COPD exacerbations: A randomized controlled cross-over trial | Wrong comparator | HFNC vs COT for hypercapnic resp failure |
| 3351 | 2020 | Pisani | Effects of high-flow nasal cannula in patients with persistent hypercapnia after an acute COPD exacerbation: a prospective pilot study | Wrong population | HFNC post-acute exacerbation |
| 2836 | 2019 | Raeisi | Value and Safety of High Flow Oxygenation in the Treatment of Inpatient Asthma: A Randomized, Double-blind, Pilot Study | Wrong population | HFNC vs COT for asthma exacerbation |
| 1831 | 2017 | Rittayamai | High-flow nasal oxygen cannula in patients with chronic obstructive pulmonary disease requiring ventilator support | Duplicate | Duplicate (#2812) |
| 2812 | 2019 | Rittayamai | Effects of high-flow nasal cannula and non-invasive ventilation on inspiratory effort in hypercapnic patients with chronic obstructive pulmonary disease: a preliminary study | Wrong study type | Prospective observational - HFNC vs NIV for hypercapnia after stabilization of AECOPD |
| 1456 | 2016 | Rittayamai | A randomized cross-over physiological study of high flow nasal oxygen cannula versus non-invasive ventilation in adult patients with cystic fibrosis: The HIFEN study | Duplicate | Duplicate (#1968) |
| 934 | 2015 | Rittayamai | Use of High-Flow Nasal Cannula for Acute Dyspnea and Hypoxemia in the Emergency Department | Wrong indication | HFNC vs COT for dyspnea and hypoxemia |
| 669 | 2014 | Rittayamai | High-flow nasal cannula versus conventional oxygen therapy after endotracheal extubation: a randomized crossover physiologic study | Wrong population | HFNC vs COT post extubation |
| 2805 | 2019 | Ruangsomboon | High-Flow Nasal Cannula Versus Conventional Oxygen Therapy in Relieving Dyspnea in Emergency Palliative Patients With Do-Not-Intubate Status: A Randomized Crossover Study | Wrong population | HFNC vs COT in palliative hypoxemic patients |
| 925 | 2015 | Saeed | Evaluation of nasal optiflow device in management of COPD patients in acute exacerbations | Wrong comparator | HFNC vs COT/VM for hypercapnic respiratory failure |
| 2012 | 2018 | Sahin | Comparison of Mask Oxygen Therapy and High-Flow Oxygen Therapy after Cardiopulmonary Bypass in Obese Patients | Wrong population | HFNC vs COT in post-extubation CABG patients |
| 656 | 2014 | Schwabbauer | Nasal high-flow oxygen therapy in patients with hypoxic respiratory failure: effect on functional and subjective respiratory parameters compared to conventional oxygen therapy and non-invasive ventilation (NIV) | Wrong indication | HFNC vs COT for hypoxic respiratory failure |
| 1991 | 2018 | Selim | Nasal High Flow Therapy Versus Oxygen Supplementation in Postoperative Care of Obstructive Sleep Apnea Patients with CPAP Non-Compliance: A Pilot Randomized Study | Wrong population | FHNC vs COT in postop OSA patients |
| 1981 | 2018 | Shebl | High-flow nasal oxygen therapy versus noninvasive ventilation in chronic interstitial lung disease patients with acute respiratory failure | Wrong indication | HFNC vs NIV for hypoxic failure in ILD patients |
| 1788 | 2017 | Sklar | A randomized cross-over physiological study of high flow nasal oxygen cannula versus non-invasive ventilation in adult patients with cystic fibrosis | Duplicate | Duplicate (#1968) |
| 1782 | 2017 | Song | The value of high-flow nasal cannula oxygen therapy after extubation in patients with acute respiratory failure | Wrong population | HFNC vs COT/VM post-extubation |
| 1781 | 2017 | Song | Clinical efficacy of high-flow nasal cannula in patients with acute exacerbation of chronic obstructive pulmonary disease | Wrong study design | Retrospective observational of HFNC for hypercapnic respiratory failure |
| 865 | 2015 | Spoletini | Better comfort and dyspnea scores with high-flow nasal cannula (HFNC) vs standard oxygen (SO) during breaks off noninvasive ventilation (NIV) | Wrong comparator | HFNC vs COT as a break for NIV |
| 1957 | 2018 | Spoletini | High-flow nasal therapy vs standard oxygen during breaks off noninvasive ventilation for acute respiratory failure: A pilot randomized controlled trial | Duplicate | Duplicate (#865) |
| 863 | 2015 | Stephan | High-Flow Nasal Oxygen vs Noninvasive Positive Airway Pressure in Hypoxemic Patients After Cardiothoracic Surgery: A Randomized Clinical Trial | Wrong population | HFNC vs COT post-extubation cardiac surgery |
| 1775 | 2017 | Stephan | High-Flow Nasal Cannula Therapy Versus Intermittent Noninvasive Ventilation in Obese Subjects After Cardiothoracic Surgery | Wrong population | HFNC vs COT for obese patients post-extubation cardiac surgery |
| 1953 | 2018 | Stoever | Safety and efficacy of high flow nasal cannula oxygen therapy in acute hypercapnic respiratory failure: A pilot study | Wrong study design | Retrospective observational HFNC vs NIV for hypercapnia |
| 2718 | 2019 | Sun | High flow nasal cannula oxygen therapy versus non-invasive ventilation for chronic obstructive pulmonary disease with acute-moderate hypercapnic respiratory failure: an observational cohort study | Wrong comparator | HFNC vs COT for hypercapnic resp failure |
| 3319 | 2020 | Tatsuishi | High-Flow Nasal Cannula Therapy With Early Extubation for Subjects Undergoing Off-Pump Coronary Artery Bypass Graft Surgery | Wrong population | HFNC vs COT post-extubation cardiac surgery |
| 1763 | 2017 | Theerawit | The efficacy of the Whispherflow CPAP system versus high flow nasal cannula in patients at high risk for postextubation failure | Wrong population | HFNC vs Whisperflow CPAP post-extubation |
| 2696 | 2019 | Thille | Effect of Postextubation High-Flow Nasal Oxygen With Noninvasive Ventilation vs High-Flow Nasal Oxygen Alone on Reintubation Among Patients at High Risk of Extubation Failure: A Randomized Clinical Trial | Wrong population | HFNC vs HFNC + NIV post-extubation |
| 140 | 2010 | Tiruvoipati | High-flow nasal oxygen vs high-flow face mask: a randomized crossover trial in extubated patients | Wrong population | Combination of HFNC and VM post extubation |
| 2685 | 2019 | Tseng | The efficacy of heated humidifier high-flow nasal cannula compared with noninvasive positive-pressure ventilation in prevention of reintubation in patients with prolonged mechanical ventilation | Wrong population | HFNC vs NIV post extubation in prolonged mechanical ventilation |
| 2683 | 209 | Twose | Comparison of high-flow oxygen therapy with standard oxygen therapy for prevention of postoperative pulmonary complications after major head and neck surgery involving insertion of a tracheostomy: a feasibility study | Wrong population | HFNC vs COT after off MV with tracheotomy |
| 3303 | 2020 | Vourc'h | High-Flow Therapy by Nasal Cannulae Versus High-Flow Face Mask in Severe Hypoxemia After Cardiac Surgery: A Single-Center Randomized Controlled Study-The HEART FLOW Study | Wrong indication | HFNC vs COT for hypoxemia after cardiac surgery |
| 3300 | 2020 | Wang | The experience of high-flow nasal cannula in hospitalized patients with 2019 novel coronavirus-infected pneumonia in two hospitals of Chongqing, China | Wrong study design | Retrospective study HFNC vs NIV for respiratory failure due to COVID |
| 2639 | 2019 | Yang | [Effect of high-flow nasal cannula oxygen therapy on diaphragmatic function in patients with acute exacerbation of chronic obstructive pulmonary disease: a prospective randomized controlled trial] | Wrong comparator | HFNC vs COT in AECOPD |
| 2634 | 2019 | Yu | Efficacy and safety of humidified high flow nasal cannula in chronic obstructive pulmonary disease complicated with type 2 respiratory failure patients after extubation: A randomized controlled trial. [Chinese] | Wrong population | HFNC vs NIV post extubation AECOPD |
| 1891 | 2018 | Zhang | [A study on the effects and safety of sequential humidified high flow nasal cannula oxygenation therapy on the COPD patients after extubation] | Wrong Population | HFNC vs NIV post extubation AECOPD |
| 1889 | 2018 | Zochios | The effect of high-flow nasal oxygen on hospital length of stay in cardiac surgical patients at high risk for respiratory complications: a randomised controlled trial | Wrong Population | HFNC vs COT in post cardiac surgery patients |
| 3659 | 2020 | Adiyeke | The Impact of High-Flow Nasal Cannula on Olfactory Function | Wrong outcomes | Impact of HFNC on olfactory function |
| 3651 | 2020 | Au | Determining the effective pre-oxygenation interval in obstetric patients using high-flow nasal oxygen and standard flow rate facemask: a biased-coin up-down sequential allocation trial | Wrong Population | Healthy obstetric patients, effect of HFNC on oxygenation |
| 2967 | 2019 | Luo | High flow nasal cannula compared with conventional oxygen therapy for acute hypoxemic respiratory failure | Wrong study design | Letter on another review |
| 1001 | 2015 | Ma | High-flow nasal cannula may save lives in acute hypoxemic respiratory failure | Wrong study design | Summary of FLORALI trial |
| 2524 | 2018 | Nagata | Domiciliary High-Flow Nasal Cannula Oxygen Therapy for Patients with Stable Hypercapnic Chronic Obstructive Pulmonary Disease. A Multicenter Randomized Crossover Trial | Wrong Population | HFNC for stable hypercapnic COPD |
| 817 | 2014 | Besch | Comparison of the alveolar recruiting effect between non-invasive mechanical ventilation and optiflow | Wrong Population | HFNC in postoperative hypoxemic respiratory failure assessing aeration |
| 3593 | 2019 | BengLeong | High flow nasal cannula oxygen versus noninvasive ventilation in adult acute respiratory failure: a systematic review of randomized-controlled trials | Wrong study design | Systematic review of HFNC vs NIV in acute respiratory failure |
| 2418 | 2018 | Hatipoglu | The use of high-flow nasal cannula during extubation | Wrong study design | Letter on another study |
| 1086 | 2015 | DelSorbo | High-Flow Nasal Cannulae or Noninvasive Ventilation for Management of Postoperative Respiratory Failure | Wrong study design | Editorial on another study |
| 3392 | 2020 | Littleton | High-Flow Nasal Cannula After Surgery-A Lateral Move From Conventional Oxygen Therapy? | Wrong study design | Comment on another study |
| 1285 | 2016 | Gupta | High flow nasal cannula (HFNC) as an alternative to noninvasive ventilation (NIV) in acute respiratory failure (ARF) in immunosuppressed patients-an Indian post liver transplant experience | Wrong Population | HFNC for postoperative hypoxic respiratory failure |
| 2445 | 2018 | Girault | High-flow nasal oxygen therapy: Recent physiological data and application to hypercapnic acute respiratory failure | Wrong study design | Review article |
| 1697 | 2016 | Attebery | High-Flow Oxygen as Noninvasive Ventilation May Complicate Timely Intubation in Patients With Acute Respiratory Distress Syndrome | Wrong study design | Letter to the editor on another study |
| 2579 | 2018 | Azoulay | High-flow nasal oxygen vs. standard oxygen therapy in immunocompromised patients with acute respiratory failure: study protocol for a randomized controlled trial | Wrong comparator | HFNC compared to COT in immunocompromised patients, study protocol |
| 2250 | 2017 | BengLeong | High flow nasal cannula oxygen versus non-invasive ventilation in acute respiratory failure? A systematic analysis of available literature | Wrong study design | Systematic review of HFNC vs NIV in acute respiratory failure |
| 3575 | 2019 | Birnbaum | Understanding the benefits of early high-flow nasal cannula therapy for adults with acute hypoxemic respiratory failure in the ED | Wrong study design | Letter to the editor on another study |
| 824 | 2014 | Beneton | Acute hypoxemia after cardiac surgery: Evaluation of a high-flow nasal cannula oxygen device. [French] | Wrong study design | Prospective observational HFNC for hypoxic respiratory failure |
| 2285 | 2018 | McKinstry | Nasal high flow therapy and PtCO_2_ in stable COPD: A randomized controlled cross-over trial | Wrong Population | HFNC in stable COPD |
| 2264 | 2017 | Azoulay | Acute hypoxemic respiratory failure in immunocompromised patients: the Efraim multinational prospective cohort study | Wrong study design | Prospective observational, interventions to prevent intubation in hypoxemic respiratory failure immunocompromised patients |
| 1356 | 2015 | Agarwal | Evaluation of the use and indications of high flow nasal therapy in adult critical care | Wrong study design | Prospective observational HFNC for respiratory failure |
| 3610 | 2019 | Azoulay | High-Flow Oxygen Therapy for Respiratory Failure in Immunocompromised Patients-Reply | Wrong study design | Letter to the editor on another study |
| 2557 | 2018 | Bocchile | The effects of high-flow nasal cannula on intubation and re-intubation in critically ill patients: a systematic review, meta-analysis and trial sequential analysis | Wrong study design | Systematic review on effect of HFNC on intubation and reintubation |
| 3570 | 2019 | Bonnevie | Nasal High Flow for Stable Patients with Chronic Obstructive Pulmonary Disease: A Systematic Review and Meta-Analysis | Wrong study design | Systematic review HFNC for stable COPD |
| 2270 | 2018 | Dodge | High velocity nasal insufflation in hypercapneic respiratory failure: Secondary analysis of randomized clinical trial | Duplicate | Duplicate (#3481) |
| 2165 | 2017 | Doshi | High velocity nasal insufflation compared to non-invasive positive pressure ventilation in the treatment of respiratory failure in the emergency department: A randomized clinical trial | Duplicate | Duplicate (#2768) |
| 2096 | 2016 | Delorme | Short-term physiological effects of nasal high flow in patients with respiratory distress. Impact of flow rates on the work of breathing | Duplicate | Duplicate (#2175) |
| 899 | 2014 | Stephan | Bilevel positive airway pressure versus optiflow in hypoxemic patients after cardiothoracic surgery (the BiPOP study): A multicenter, randomized, noninferiority, open trial | Duplicate | Duplicate (#863) |
| 3564 | 2019 | Bruni | High Flow Through Nasal Cannula in Stable and Exacerbated Chronic Obstructive Pulmonary Disease Patients | Wrong study design | Review article |
| 838 | 2015 | Yildirim | High-flow nasal oxygen vs noninvasive positive airway pressure in hypoxemic patients after cardiothoracic surgery: A randomized clinical trial. [Turkish] | Wrong study design | Comment on another study |
| 902 | 2014 | Sowho | Usability of high flow therapy in COPD patients | Wrong Population | HFNC for chronic hypercapnia in COPD |
| 1476 | 2017 | Nishimura | High-flow nasal cannula is superior to noninvasive ventilation to prevent reintubation? | Wrong study design | Editorial letter |
| 2523 | 2018 | Nakamura | Highflow nasal cannula therapy has the effectiveness for acute respiratory failure including hypercapnic status: a single-centered retrospective study | Wrong outcomes | HFNC for respiratory failure, retrospective study, limited outcomes reported |
| 2079 | 2018 | Patel | Nasal high-flow oxygen therapy for sleep-related hypoventilation in acute on chronic respiratory failure | Wrong study design | Case report of HFNC for acute on chronic hypercapnic respiratory failure |
| 1631 | 2017 | Hernandez | Preventing reintubation: Role of stratification of high-risk for reintubation in the selection of appropriate therapy a post HOC analysis | Wrong study design | Posthoc analysis of an excluded trial (#1267) - HFNC vs NIV post-extubation |
| 2508 | 2018 | Omote | High-Flow nasal cannula therapy for acute respiratory failure in patients with interstitial pneumonia: A retrospective observational study | Wrong study design | Retrospective study of HFNC for interstitial pneumonia |
| 413 | 2013 | Nilius | Nasal high flow oxygen therapy attenuates nocturnal hypoventilation in COPD patients with hypercapnic respiratory failure | Wrong Population | HFNC for chronic hypercapnia in COPD |
| 2656 | 2019 | Wang | [Progress in the application of high-flow nasal cannula oxygenation in immunosuppressed patients with acute respiratory failure] | Wrong study design | Review of the role of HFNC in immunosuppressed patients with respiratory failure |
| 2123 | 2016 | Cadier | High-Flow vs Conventional Oxygen Therapy and Risk of Reintubation | Wrong study design | Letter about another study |
| 1052 | 2015 | Girault | High-Flow Nasal Oxygen Therapy for Postextubation Acute Hypoxemic Respiratory Failure | Wrong study design | Letter to the editor on another study |
| 1525 | 2017 | MartinsTomazini | High-Flow Oxygen vs Noninvasive Ventilation for Postextubation Respiratory Failure | Wrong study design | Letter to the editor on another study |
| 1087 | 2015 | Curley | Noninvasive respiratory support for acute respiratory failure-high flow nasal cannula oxygen or non-invasive ventilation? | Wrong study design | Editorial letter |
| 3005 | 2019 | Lazo | High Flow Versus Standard Oxygen Therapy After Noninvasive Ventilation Withdrawal | Wrong study design | Letter to the editor |
| 1192 | 2016 | Lepere | High-flow nasal cannula oxygen supply as treatment in hypercapnic respiratory failure | Wrong study design | Case report of HFNC for acute hypercapnic respiratory failure |
| 2998 | 2019 | Leung | Comparison of high-flow nasal cannula versus oxygen face mask for environmental bacterial contamination in critically ill pneumonia patients: a randomized controlled crossover trial | Wrong outcomes | Assessing environmental contamination with HFNC vs COT |
| 1741 | 2017 | Watler | Postextubation High-Flow Nasal Cannula Oxygen, Randomized Trial of an ICU Quality Improvement Intervention, and Midodrine during Recovery from Septic Shock | Wrong study design | Letter to the editor on another study |
| 180 | 2010 | Hernandez | Noninvasive ventilation reduces intubation in chest trauma-related hypoxemia: a randomized clinical trial | Wrong intervention | Facemask vs NIV in trauma associated hypoxemia |
| 47 | 2006 | Woodhead | Comparing two methods of delivering high-flow gas therapy by nasal cannula following endotracheal extubation: a prospective, randomized, masked, crossover trial | Wrong Population | Pediatric population |
| 3630 | 2020 | Crimi | High Flow Nasal Therapy Use in Patients with Acute Exacerbation of COPD and Bronchiectasis: A Feasibility Study | Duplicate | Duplicate ( #2188) |
| 2843 | 2019 | Prieur | Acute effects of nasal high-flow during exercise in COPD patients after an exacerbation: a randomized controlled cross-over trial | Wrong Population | HFNC for patients now stable after AECOPD |
| 2550 | 2018 | Braunlich | Effectiveness of nasal high flow in hypercapnic COPD patients is flow and leakage dependent | Wrong Population | HFNC in stable hypercapnic respiratory failure |
| 3120 | 2019 | Jabbari | Clinical usage of high-flow oxygenation in postcardiac surgery patients | Wrong study design | Letter to the editor |
| 2465 | 2018 | Francois | High flow nasal oxygen therapy for management acute hypercapnic respiratory failure in emergency department | Wrong study design | Observational study of HFNC for hypercapnic respiratory failure |
| 3569 | 2019 | Branick | Use of nasal high flow oxygen on the general medical floor can prevent ICU admission and escalation of care in COPD patients | Wrong study design | Observational study of HFNC for hypoxic respiratory failure in COPD patients |
| 1170 | 2016 | Mauri | Correlation between improvement of physiological variables and increasing flow rates during High Flow Nasal Cannula (HFNC) therapy | Wrong comparator | Comparing different HFNC settings in hypoxic respiratory failure |
| 3493 | 2019 | DiMauro | High-flow nasal canula not noninferior to nasal CPAP | Wrong Population | Pediatric population |
| 2781 | 2018 | David | Treatments and outcomes of hypoxemia in immunosuppressed patients compare to immunocompetent patients in French speaking ICU: A sub-study from the SPECTRUM study | Wrong outcomes | NIV vs HFNC in hypoxemic patients, immunosuppressed population |
| 2640 | 2019 | Yang | Evaluation of the effect of two active warming and humidifying high-flow oxygen therapy systems in patients with tracheotomy | Wrong Population | Tracheostomized patients, comparing humidified and non-humidified HFNC |
| 2467 | 2018 | Fingleton | Feasibility of NHF for acute hypercapnic respiratory failure in COPD | Wrong outcomes | Comparing rates of ABG sampling in patients with hypercapnic respiratory failure |
| 1952 | 2018 | Storgaard | Long-term effects of oxygen-enriched high-flow nasal cannula treatment in COPD patients with chronic hypoxemic respiratory failure | Wrong Population | HFNC in patients with chronic hypoxemia in COPD |
| 2013 | 2013 | Hui | High-flow oxygen (HFO) and bilevel positive airway pressure (BiPAP) for refractory dyspnea in patients with advanced cancer: A randomized controlled trial | Wrong Population | HFNC vs NIV in palliative setting for refractory dyspnea |
| 2134 | 2016 | Braunlich | Nasal highflow improves ventilation in patients with COPD | Wrong Population | HFNC for hypercapnia in stable COPD |
| 810 | 2014 | Brotfain | Comparison of the effectiveness of high flow nasal oxygen cannula vs. standard non-rebreather oxygen face mask in post-extubation intensive care unit patients | Wrong study design | Observational study HFNC vs COT post-extubation |
| 561 | 2011 | Corley | Oxygen delivery through high-flow nasal cannulae increase end-expiratory lung volume and reduce respiratory rate in post-cardiac surgical patients | Wrong study design | Observational study of HFNC in post-cardiac surgery patients |
| 1845 | 2017 | Prieur | Effect of high-flow nasal therapy during acute aerobic exercise in patients with chronic obstructive pulmonary disease after exacerbation: protocol for a randomised, controlled, cross-over trial | Wrong Population | HFNC Stable chronic hypercapnic respiratory failure after recovery from an extubation (remote) |
| 2760 | 2018 | Eaton Turner | Cost-effectiveness analysis of the use of high-flow oxygen through nasal cannula in intensive care units in NHS England | Wrong study design | Cost-effectiveness of HFNC in ICUs |
| 1937 | 2018 | Thille | High-flow nasal cannula oxygen therapy alone or with non-invasive ventilation during the weaning period after extubation in ICU: the prospective randomised controlled HIGH-WEAN protocol | Duplicate | Duplicate (#2696) |
| 193 | 2010 | Austin | High flow oxygen increases mortality in COPD patients in a pre-hospital setting: A RCT | Duplicate | Duplicate (#192) |
| 1683 | 2017 | Anonymous | Erratum: Effect of postextubation high-flow nasal cannula vs noninvasive ventilation on reintubation and postextubation respiratory failure in high-risk patients: A randomized clinical trial (JAMA - Journal of the American Medical Association (2016) 316:15 (1565-1574)) | Wrong study design | Correction note for a trial |
| 3162 | 2019 | Haywood | HVNI vs NIPPV in the treatment of acute decompensated heart failure: Subgroup analysis of a multi-center trial in the ED | Duplicate | Duplicate (#2414) |
| 2195 | 2017 | Corley | High-flow oxygen via tracheostomy improves oxygenation in patients weaning from mechanical ventilation: a randomised crossover study | Wrong Population | HFNC for tracheostomized patients weaning from mechanical ventilation |
| 231 | 2011 | Murphy | A randomised controlled study suggesting equivalence between full face vs. nasal high flow oxygen delivery on health outcomes | Wrong study design | Review of another study |
| 2112 | 2016 | Cirio | Effects of heated and humidified high flow gases during high-intensity constant-load exercise on severe COPD patients with ventilatory limitation | Wrong Population | HFNC for patients with stable hypercapnia |
| 1863 | 2017 | Pavlov | Nasal high-flow therapy for type II respiratory failure in COPD: A report of four cases | Wrong study design | Case series HFNC for hypercapnic respiratory failure |
| 3361 | 2020 | Nori | Can High-Flow Oxygen Overstep Low-Flow Oxygen Therapy in Weaning From Noninvasive Ventilation in Patients With Acute Respiratory Failure Due to Chronic Obstructive Pulmonary Disease Exacerbation? | Wrong study design | Letter to the editor |
| 1227 | 2016 | Kim | Can a high-flow nasal cannula substitute for noninvasive positive pressure ventilation in post-extubation respiratory failure? | Wrong study design | Narrative review HFNC in post-extubation setting |
| 2966 | 2019 | Luo | High-flow nasal cannula oxygen therapy versus conventional oxygen therapy in patients after planned extubation | Wrong study design | Letter about another study |
| 2311 | 2018 | Long | Is High-Flow Nasal Cannula More Effective Than Conventional Oxygen Therapy for Preventing Escalation of Respiratory Support in Patients With Acute Respiratory Failure? | Wrong study design | Review of another study |
| 962 | 2015 | Ntoumenopoulos | In acute exacerbation of chronic obstructive pulmonary disease, the use of low-flow oxygen therapy and not high-flow saves lives | Wrong study design | Commentary |
| 228 | 2011 | Ntoumenopoulos | Using titrated oxygen instead of high flow oxygen during an acute exacerbation of chronic obstructive pulmonary disease (COPD) saves lives | Wrong study design | Review of another study |
| 3213 | 2019 | Frat | High-Flow Oxygen Therapy for Respiratory Failure in Immunocompromised Patients | Wrong study design | Letter to the editor |
| 2461 | 2018 | Frat | Predictors of Intubation in Patients With Acute Hypoxemic Respiratory Failure Treated With a Noninvasive Oxygenation Strategy | Wrong outcomes | Posthoc analysis of an RCT, assessing intubation predictors |
| 2744 | 2019 | Segovia | Combination Therapy in Patients with Acute Respiratory Failure: High-Flow Nasal Cannula and Non-Invasive Mechanical Ventilation | Wrong study design | Case study |
| 3468 | 2020 | Fiorentino | Unconventional use of high-flow nasal cannula in acute exacerbation of idiopathic pulmonary fibrosis with high levels of hypercapnia | Wrong study design | Case study |
| 1886 | 2017 | Achaiah | Does enhanced respiratory support affect clinical outcomes in ILD Patients with acute Respiratory failure? | Wrong study design | Retrospective study outcomes for ILD patients and acute respiratory failure |
| 1737 | 2017 | Weinreich | Long term high flow heated oxygen treatment in COPD-lung function and physical ability | Wrong Population | HFNC for chronic hypercapnia in COPD |
| 1736 | 2016 | Weinreich | Long-term nasal high flow treatment with oxygen in COPD-exacerbations, admissions and mortality | Wrong Population | HFNC for chronic hypercapnia in COPD |
| 2573 | 2018 | Barneche | High flow nasal cannula improves exercise capacity in COPD patients: Crossover trial | Wrong Population | HFNC for chronic hypercapnia in COPD |
| 324 | 2012 | Cleven | Effect of transnasal "high-flow oxygen insufflation" in patients with severe COPD | Wrong Population | HFNC for chronic hypercapnia in COPD |
| 2188 | 2017 | Crimi | High-flow oxygen therapy for treatment of acute exacerbation of copd with bronchiectasis | Wrong study design | HFNC for AECOPD, no control group |
| 2421 | 2018 | Hanci | High flow nasal oxygen therapy in patients with acute exacerbations of COPD | Wrong study design | HFNC for AECOPD without hypercapnia |
| 805 | 2014 | Canovas | Comparison between high-flow devices with active oxygen humidification and ventilation not invasive in hypoxemic respiratory failure | Wrong Population | Case control of HFNC and NIV for hypoxic respiratory failure |
| 1849 | 2017 | Plotnikow | High-flow nasal cannula oxygen for reverting severe acute exacerbation of chronic obstructive pulmonary disease: A case report | Wrong study design | Case study |
| 1013 | 2015 | Lee | Feasibility of high-flow nasal cannula oxygen therapy for acute respiratory failure in patients with hematologic malignancies: A retrospective single-center study | Wrong study design | Prospective observational of HFNC for respiratory failure in pts with hematologic malignancies |
| 2961 | 2019 | Macari | High-flow oxygen therapy vs non invasive ventilation: A prospective randomized cross-over physiological study of alveolar recruitment in acute respiratory failure | Wrong Population | Randomized crossover of alveolar recruitment in hypoxemic respiratory failure |
| 1242 | 2016 | Jones | High-flow nasal cannula is superior to conventional oxygen delivery after extubation | Wrong study design | Review of another study |
| 2468 | 2018 | Fimognari | High-flow nasal cannula oxygen therapy for acute respiratory failure in a non-intensive geriatric setting | Wrong study design | Case series |
| 230 | 2011 | Nicolet | [High-flow nasal oxygen for severe hypoxemia after cardiac surgery] | Wrong study design | Prospective observational of HFNC for hypoxic respiratory failure |
| 3516 | 2019 | Cortegiani | High-flow nasal therapy versus noninvasive ventilation in COPD patients with mild-to-moderate hypercapnic acute respiratory failure: study protocol for a noninferiority randomized clinical trial | Duplicate | Duplicate (#4445) |
| 2773 | 2018 | DiMussi | High-flow nasal cannula oxygen therapy decreases postextubation neuroventilatory drive and work of breathing in patients with chronic obstructive pulmonary disease | Wrong study design | Non-randomized crossover trial of HFNC post-extubation in COPD patients for work of breathing assessment |
| 2456 | 2018 | Fulton | High flow nasal oxygen after bariatric surgery (OXYBAR), prophylactic post-operative high flow nasal oxygen versus conventional oxygen therapy in obese patients undergoing bariatric surgery: study protocol for a randomised controlled pilot trial | No results published | No results published |
| 6239 | 2021 | Ruangsomboon | Nasal High Flow Oxygen Versus Non Invasive Positive Pressure Ventilation in Emergency Department Patients With Cardiogenic Pulmonary Edema: a Randomized Non-inferiority Trial | No results published | No results published |
| 5009 | 2021 | Plotnikow | Erratum: High-Flow Oxygen Therapy Application in Chronic Obstructive Pulmonary Disease Patients With Acute Hypercapnic Respiratory Failure: A Multicenter Study: Erratum | Wrong study design | Observational case series |
| 6379 | 2021 | Xu | Sequential treatment of chronic obstructive pulmonary disease concurrent with respiratory failure by high-flow nasal cannula therapy | Wrong Population | Post-extubation |
| 5695 | 2021 | Long | High flow nasal cannula in acute exacerbation of chronic obstructive pulmonary disease | Wrong study design | Letter to the editor |
| 1064 | 2015 | Frat | Sequential application of oxygen therapy via high-flow nasal cannula and noninvasive ventilation in acute respiratory failure: an observational pilot study | Wrong study design | Observational pilot study |
| 2106 | 2016 | Coudroy | High-flow oxygen therapy through a nasal cannula versus noninvasive ventilation versus in immunocompromised patients with acute respiratory failure | Wrong study design | Observational study |
| 3514 | 2019 | Coudroy | High-flow nasal oxygen therapy alone or with non-invasive ventilation in immunocompromised patients admitted to ICU for acute hypoxemic respiratory failure: the randomised multicentre controlled FLORALI-IM protocol | No results published | No results published |
| 485 | 2013 | Futier | The OPERA trial - comparison of early nasal high flow oxygen therapy with standard care for prevention of postoperative hypoxemia after abdominal surgery: study protocol for a multicenter randomized controlled trial | Wrong outcomes | HFNC vs COT for hypoxemia post extubation |
| 3417 | 2020 | Koga | Comparison of high-flow nasal cannula oxygen therapy and non-invasive ventilation as first-line therapy in respiratory failure: a multicenter retrospective study | Wrong study design | Retrospective study |
| 3483 | 2020 | Ding | Efficacy and safety of early prone positioning combined with HFNC or NIV in moderate to severe ARDS: a multi-center prospective cohort study | Wrong intervention | Effect of prone positioning |
| 1312 | 2016 | Emlet | High-Flow Oxygen through Nasal Cannula in Acute Hypoxemic Respiratory Failure: The FLORALI study | Wrong study design | Letter to the editor |
| 1520 | 2017 | Mauri | Setting high flow nasal cannula to maximize physiologic benefits | Duplicate | Duplicate (#1519) |
| 1789 | 2017 | Sklar | A randomized cross-over physiological study of high flow nasal oxygen cannula versus non-invasive ventilation in adult patients with cystic fibrosis: The HIFEN study | Duplicate | Duplicate (31968) |
| 2976 | 2019 | Lockstone | Non-Invasive Positive airway Pressure thErapy to Reduce Postoperative Lung complications following Upper abdominal Surgery (NIPPER PLUS): protocol for a single-centre, pilot, randomised controlled trial | No results published | Protocol only |
| 881 | 2014 | Yuko | Nasal high-flow oxygen therapy vs non-invasive ventilation for postoperative respiratory failure | Wrong study design | Observational study |
| 613 | 2012 | Rittayamai | Benefits of high flow nasal oxygen cannula therapy after endotracheal extubation | Duplicate | Duplicate (#669) |
| 2029 | 2018 | Ricard | Comparison of high flow nasal cannula oxygen and conventional oxygen therapy on ventilatory support duration during acute-on-chronic respiratory failure: study protocol of a multicentre, randomised, controlled trial. The 'HIGH-FLOW ACRF' study | No results published | Protocol only |
| 3230 | 2019 | Fakharian | Value and safety of high flow oxygenation in the treatment of inpatient asthma, a randomized, double-blind, clinical trial | Wrong Population | HFNC for acute asthma exacerbation |
| 2582 | 2018 | Attaway | Safety and efficacy of oxygen delivery via high flow nasal cannula (HFNC) in patients with structural lung disease and chronic respiratory failure | Wrong Population | Chronic respiratory failure |
| 2160 | 2017 | Dumas | Initial ventilation strategy and risk for intubation in immunocompromised patients with acute respiratory failure | Wrong study design | Observational study |
| 995 | 2015 | Mauri | Effects of high-flow nasal cannula therapy on oxygenation, lung volumes and CO<inf>2</inf> removal in critically ill hypoxemic patients: Preliminary results | Duplicate | Duplicate (#1519) |
| 578 | 2011 | Atwood | Effect of high flow highly humidified air via nasal cannula on respiratory effort in patients with advanced COPD | Wrong Population | HFNC for chronic, stable COPD |
| 2266 | 2017 | Atwood | Impact of Heated Humidified High Flow Air via Nasal Cannula on Respiratory Effort in Patients with Chronic Obstructive Pulmonary Disease | Duplicate | Same study as #578 |
| 3198 | 2019 | Gaspari | Use of high-flow nasal cannula vs standard oxygen therapy via venturi mask in liver transplantation after extubation to prevent the hypoxemia: A matched-controlled study | Wrong study design | Observational, matched control study |
| 2782 | 2018 | D'Espiney | Critical care extubation in Type II respiratory failure with nasal high flow therapy | Wrong study design | Retrospective observational study of HFNC vs NIV post extubation |
| 3568 | 2018 | Braunlich | Nasal high-flow versus noninvasive ventilation in patients with chronic hypercapnic COPD | Wrong Population | HFNC for chronic, stable COPD |
| 2761 | 2018 | Dumas | Oxygenation/non-invasive ventilation strategy and risk for intubation in immunocompromised patients with hypoxemic acute respiratory failure | Wrong study design | Retrospective database analysis |
| 2414 | 2018 | Haywood | High velocity nasal insufflation vs noninvasive positive pressure ventilation in heart failure | Wrong Population | HFNC vs NIV in heart failure |
| 2936 | 2019 | Mckinstry | Nasal high-flow therapy compared with non-invasive ventilation in COPD patients with chronic respiratory failure: A randomized controlled cross-over trial | Wrong Population | HFNC for chronic, stable COPD |
| 175 | 2010 | Idone | Nasal high-flow oxygen therapy vs. standard oxygen therapy via venturi mask after extubation: Preliminary results of a randomized, controlled trial | Duplicate | Duplicate (#714) |
| 2549 | 2018 | Braunlich | Nasal high-flow in acute hypercapnic exacerbation of COPD | Wrong study design | Non-randomized observational study |
| 3843 | 2020 | Remy | High-flow nasal cannula may be no safer than non-invasive positive pressure ventilation for COVID-19 patients | Wrong study design | Letter to the editor |
| 4043 | 2015 | Coudroy | High-flow nasal cannula oxygen therapy versus noninvasive ventilation versus in immunocompromised patients with acute respiratory failure | Wrong study design | Retrospective observational study |
| 4713 | 2020 | Miller | High-Flow Nasal Cannula Use in the Management of Acute Hypercapnic Respiratory Failure Due to Cardiogenic Pulmonary Edema: Heavyweight or Light Heavyweight? | Wrong study design | Editorial |
| 4067 | 2019 | Anonymous | 44th Annual Meeting of the European Society for Blood and Marrow Transplantation: Physicians Award Winners | Wrong intervention | No HFNC studies identified in abstracts |
| 4303 | 2020 | James | Non-invasive ventilation for acute respiratory failure (in COVID-19 patients): the non-ending story? | Wrong study design | Commentary |
| 3981 | 2020 | Despres | Prone positioning combined with high-flow nasal or conventional oxygen therapy in severe Covid-19 patients | Wrong comparator | HFNC vs COT |
| 4421 | 2020 | Demoule | High-Flow Nasal Cannula in Critically III Patients with Severe COVID-19 | Wrong study design | Retrospective chart review |
| 4919 | 2020 | Shanqun | Clinical therapeutic effect of high flow oxygen inhalation (hfnc) combined with respiratory rehabilitation on patients with acute attack of COPD: a multicenter prospective randomized controlled clinical study | No results published | Study in progress |
| 4438 | 2020 | Curtis | High-flow nasal cannula can't be considered non-inferior to noninvasive ventilation in patients with chronic obstructive pulmonary disease who develop respiratory failure after extubation | Wrong study design | Letter to the editor |
| 4920 | 2020 | He | High-flow nasal oxygen versus NIV in solid organ transplantation patients with acute respiratory failure: a randomized controlled trial | No results published | Study in progress |
| 4005 | 2020 | Casey | Effect of protocolized post-extubation respiratory support on reintubation: A randomized clinical trial | Wrong Population | Post-extubation intervention |
| 4892 | 2020 | Bhardwaj | Comparing two oxygen delivery techniques in patients with breathing difficulty | No results published | Not yet recruiting |
| 1724 | 2017 | Yu | Effect of High-Flow Nasal Cannula versus Conventional Oxygen Therapy for Patients with Thoracoscopic Lobectomy after Extubation | Wrong Population | HFNC vs COT Post extubation after lobectomy |
| 3956 | 2020 | Geng | High flow nasal cannula is a good treatment option for COVID-19 | Wrong study design | Case series HFNC for COVID-19 |
| 3953 | 2020 | Gonzalez-Castro | High-flow oxygen therapy with spontaneous breathing prono position in SARS-CoV-2 pneumonia | Wrong study design | Case report HFNC for COVID-19 |
| 3917 | 2020 | Lalla | The utility of high-flow nasal cannula oxygen therapy in the management of respiratory failure secondary to COVID-19 pneumonia | Wrong study design | Letter to the Editor HFNC for COVID-19 |
| 2089 | 2018 | Miyakawa | The role of high flow nasal oxygen in the immunocompromised patient with acute hypoxemic respiratory failure | Wrong study design | Retrospective chart review HFNC for acute respiratory failure |
| 3848 | 2020 | Rali | High-flow Nasal Cannula Oxygenation Revisited in COVID-19 | Wrong study design | Letter to the Editor, Case report HFNC for COVID-19 |
| 3798 | 2020 | Tu | Prone positioning in high-flow nasal cannula for COVID-19 patients with severe hypoxemia: a pilot study | Wrong study design | Letter to the Editor, prospective observational HFNC for COVID-19 |
| 3790 | 2020 | Vianello | High-flow nasal cannula oxygen therapy to treat patients with hypoxemic acute respiratory failure consequent to SARS-CoV-2 infection | Wrong study design | Prospective observational HFNC for COVID-19 |
| 3778 | 2020 | Xu | Early awake prone position combined with high-flow nasal oxygen therapy in severe COVID-19: a case series | Wrong study design | Case series, HFNC for COVID-19 |

**e-Table 4:** Risk of Bias Table

|  |  |  |  |
| --- | --- | --- | --- |
| Low risk of bias | Probably low risk of bias | Probably high risk of bias | High risk of bias |

| **Mortality** | **Random Sequence Generation** | **Allocation Concealment** | **Blinding of participants and personnel** | **Blinding of outcome assessors** | **Incomplete outcome data** | **Selective reporting** | **Other Bias** |
| --- | --- | --- | --- | --- | --- | --- | --- |
| Lee | Probably Low  (Randomization performed but method not described) | Probably low (Method of allocation concealment not documented but baseline characteristics are evenly distributed between control and intervention) | Probably low (blinding not described but objective outcome) | Probably low (Ethics section states that data was “treated anonymously during analysis”) | High (Greater than 10% dropout rate following randomization that was not accounted for during analysis - per-protocol analysis performed) | Probably high (no description of protocol publication, blood gas endpoints are reported but not outlined in methods) | Probably low (no funding or COI reported but study reporting unclear on methodology with randomization of patients performed but study described as an observational trial) |
| Wang | Probably low  (Randomization performed but method not described in available abstract) | Probably low  (Method of allocation concealment not described in available abstract) | Probably low  (Blinding not possible due to nature of intervention but mortality is an objective outcome) | Probably low  (Not discussed in available abstract but mortality is an objective outcome) | Probably low  (ITT not described in available abstract) | Probably low  (Trial registration and predetermined outcomes not described in available abstract) | Probably low  (No funding reported in available abstract) |
| Papachatzakis | Probably low  (Stated “Randomized”, but method not reported) | Probably low  (Method of allocation concealment not documented, but baseline characteristics fairly even between intervention and control group) | Probably low (Not possible to blind NIV vs HFNC, but objective outcome) | Probably low (Not discussed however mortality is an objective outcome) | Probably low (Did not define if analysis was ITT, but no reported loss of patients) | Probably low (Did not describe trial registration, however a trial protocol was submitted a priori to their ethics board) | Low  (No funding or COI reported) |
| Cortegiani | Low  (Computer generated randomization sequence) | Low  (Sequentially numbered, sealed, opaque envelopes) | Probably low (Patients and personnel not blinded however mortality is an objective outcome) | Probably low (Data collectors were not blinded, however statisticians were blinded and mortality is an objective outcome) | Probably low  (ITT and per-protocol analysis reported, very low loss to follow-up) | Low (All pre-specified outcomes reported, protocol published and trial registered on clinical trials.gov) | Low  (No funding, some device support from Fisher and Paykel however it was stated they played no role in protocol design, data management, interpretation or analysis) |
| **Intubation** | Random Sequence Generation | Allocation Concealment | Blinding of participants and personnel | Blinding of outcome assessors | Incomplete outcome data | Selective reporting | Other Bias |
| Lee | Probably Low  (Randomization performed but method not described) | Probably low (Method of allocation concealment not documented but baseline characteristics are evenly distributed between control and intervention) | Probably low (blinding not described but objective outcome) | Probably low (Ethics section states that data was “treated anonymously during analysis”) | High (Greater than 10% dropout rate following randomization that was not accounted for during analysis - per-protocol analysis performed) | Probably high (no description of protocol publication, blood gas endpoints are reported but not outlined in methods) | Probably low (no funding or COI reported but study reporting unclear on methodology with randomization of patients performed but study described as an observational trial) |
| Wang | Probably low  (Randomization performed but method not described in available abstract) | Probably low  (Method of allocation concealment not described in available abstract) | Probably low  (Blinding not possible due to nature of intervention but intubation is an objective outcome) | Probably low  (Not discussed in available abstract but intubation is an objective outcome) | Probably low  (ITT not described in available abstract) | Probably low  (Trial registration and predetermined outcomes not described in available abstract) | Probably low  (No funding reported in available abstract) |
| Doshi | Low (Computer generated randomization schedule) | Probably low  (Sealed, sequentially numbered, envelopes-did not mention if opaque) | Probably low (Patients and personnel not blinded however intubation is an objective outcome) | Probably low (Did not describe if outcome assessors were blinded to intervention, however intubation is an objective outcome) | Definitely no  (ITT analysis with pre-specified plan for analysis of crossovers, no loss to follow-up) | Low (All pre-specified outcomes were reported and trial was registered) | Probably high (Vapotherm was involved with trial design, selection of sites, and site management) |
| Cortegiani | Low  (Computer generated randomization sequence) | Low (Sequentially numbered, sealed, opaque envelopes) | Probably low (Patients and personnel not blinded however intubation is an objective outcome) | Probably low (Research collectors were not blinded, however statisticians were blinded and intubation is an objective outcome) | Probably low (ITT and per-protocol analysis reported, very low loss to follow-up | Low (All pre-specified outcomes reported, protocol published and trial registered on clinical trials.gov) | Low (No funding, some device support from Fisher and Paykel however it was stated they played no role in protocol design, data management, interpretation or analysis) |
| **ICU LOS** | Random Sequence Generation | Allocation Concealment | Blinding of participants and personnel | Blinding of outcome assessors | Incomplete outcome data | Selective reporting | Other Bias |
| Wang | Probably low  (Randomization performed but method not described in available abstract) | Probably low  (Method of allocation concealment not described in available abstract) | Probably low  (Blinding not possible due to nature of intervention but length of stay is an objective outcome) | Probably low  (Not discussed in available abstract but length of stay is an objective outcome) | Probably low  (ITT not described in available abstract) | Probably low  (Trial registration and predetermined outcomes not described in available abstract) | Probably low  (No funding reported in available abstract) |
| Doshi | Low (Computer generated randomization schedule) | Probably low  (Sealed, sequentially numbered, envelopes-did not mention if opaque) | Probably low (Patients and personnel not blinded however LOS is an objective outcome) | Probably low (Did not describe if outcome assessors were blinded to intervention, however LOS is an objective outcome) | Low  (ITT analysis with pre-specified plan for analysis of crossovers, no loss to follow-up) | Low (All pre-specified outcomes were reported and trial was registered) | Probably high (Vapotherm was involved with trial design, selection of sites, and site management) |
| **Hospital LOS** | Random Sequence Generation | Allocation Concealment | Blinding of participants and personnel | Blinding of outcome assessors | Incomplete outcome data | Selective reporting | Other Bias |
| Cong | Probably low  (Randomization planned but method not described) | Probably low (Allocation concealment not documented, but baseline characteristics fairly even) | Probably low (Cannot blind patients/personnel but LOS is an objective outcome) | Probably low (Described as single blind, statisticians most likely group to be blinded; LOS is an objective outcome) | Probably low (Unknown if analysis ITT or per-protocols, but no reported loss to follow-up) | Probably low (No documented trial registration but protocol was approved by the local ethics committee) | Low (No funding or COI reported). |
| Cortegiani | Low  (Computer generated randomization sequence) | Low  (Sequentially numbered, sealed, opaque envelopes) | Probably low (Patients and personnel not blinded however LOS is an objective outcome) | Probably low (Research collectors were not blinded, however statisticians were blinded and LOS is an objective outcome) | Probably low  (ITT and per-protocol analysis reported, very low loss to follow-up) | Low (All pre-specified outcomes reported, protocol published and trial registered on clinical trials.gov) | Low  (No funding, some device support from Fisher and Paykel however it was stated they played no role in protocol design, data management, interpretation or analysis) |
| Doshi | Low (Computer generated randomization schedule) | Probably low  (Sealed, sequentially numbered, envelopes-did not mention if opaque) | Probably low (Patients and personnel not blinded however LOS is an objective outcome) | Probably low (Did not describe if outcome assessors were blinded, however LOS is an objective outcome) | Low  (ITT analysis with pre-specified plan for analysis of crossovers, no loss to follow-ups) | Low (All pre-specified outcomes were reported and trial was registered) | Probably high (Vapotherm was involved with trial design, selection of sites, and site management) |
| Papachatzakis | Probably low  (Stated “Randomized” but method not reported) | Probably low  (Method of allocation concealment not documented, but baseline characteristics fairly even) | Probably low (Not possible to blind NIV vs HFNC, but objective outcome) | Probably low (Not discussed however LOS is an objective outcome) | Probably low (Did not define if ITT, but no reported loss of patients). | Probably low (did not describe trial registration, however a trial protocol was submitted a priori to the local research ethics boards) | Low  (No funding or COI reported) |
| **Comfort** | Random Sequence Generation | Allocation Concealment | Blinding of participants and personnel | Blinding of outcome assessors | Incomplete outcome data | Selective reporting | Other Bias |
| Sklar | Probably low (Patients enrolled in random order but method of randomization not described) | Probably low (Opaque, sealed envelope but not sequential numbering not described) | High (Patients and personnel not blinded, comfort is a subjective outcome) | High (No blinding described, comfort is a subjective outcome) | Low  (Crossover trial with no loss to follow up in comfort outcome) | High (All pre-specified outcomes reported, but comfort and dyspnea outcomes added; trial registered on clinicaltrials.gov) | Probably high (there was industry funding to complete the study, role in trial design and implementation not described) |
| Cortegiani | Low  (Computer generated randomization sequence) | Low (Sequentially numbered, sealed, opaque envelopes) | High (Patients and personnel not blinded, comfort is a subjective outcome) | High (Research collectors were not blinded, comfort is a subjective outcome) | Probably low (ITT and per-protocol analysis reported, very low loss to follow-up) | Low (All pre-specified outcomes reported, protocol published and trial registered on clinical trials.gov) | Low (No funding, some device support from Fisher and Paykel however it was stated they played no role in protocol design, data management, interpretation or analysis |
| **Dyspnea** | Random Sequence Generation | Allocation Concealment | Blinding of participants and personnel | Blinding of outcome assessors | Incomplete outcome data | Selective reporting | Other Bias |
| Cortegiani | Low  (Computer generated randomization sequence) | Low (Sequentially numbered, sealed, opaque envelopes) | High (Patients and personnel not blinded, dyspnea is a subjective outcome) | High (Research collectors were not blinded, dyspnea is a subjective outcome) | Probably low (ITT and per-protocol analysis reported, very low loss to follow-up) | Low (All pre-specified outcomes reported, protocol published and trial registered on clinical trials.gov) | Low (No funding, some device support from Fisher and Paykel however it was stated they played no role in protocol design, data management, interpretation or analysis) |
| Doshi | Low (Computer generated randomization schedule) | Probably low  (Sealed, sequentially numbered, envelopes-did not mention if opaque) | High (Patients and personnel not blinded; dyspnea is a subjective outcome) | Probably high (Did not describe if outcome assessors were blinded to intervention, dyspnea is a subjective outcome) | Definitely low  (ITT analysis with pre-specified plan for analysis of crossovers, no loss to follow-up) | Low (All pre-specified outcomes were reported and trial was registered) | Probably high (Vapotherm was involved with trial design, selection of sites, and site management) |
| Rezaei | Low (Computer generated randomization) | Probably low (Allocation concealment not described; baseline characteristics fairly similar, baseline PaCO2 higher in group B but does not reach significance) | High (Patients and personnel not blinded; dyspnea is a subjective outcome) | Probably low (States investigators and data analyzers blinded, presuming investigators assessed the outcomes, risk of bias low) | Probably low (crossover trial, no losses to follow-up) | High (All pre-specified outcomes reported but more added from trial registration; trial registered on Iranian clinical trial registry) | Low (No COI or funding reported) |
| Sklar | Probably low (Patients enrolled in random order but method of randomization not described) | Probably low (Opaque, sealed envelope but not sequential numbering not described) | High (Patients and personnel not blinded, dyspnea is a subjective outcome) | High (No blinding described, dyspnea is a subjective outcome) | Low  (Crossover trial with no loss to follow up in dyspnea outcome) | High (All pre-specified outcomes reported, but comfort and dyspnea outcomes added; trial registered on clinicaltrials.gov) | Probably high (There was industry funding to complete the study, role in trial design and implementation not described) |
| **PO2** | Random Sequence Generation | Allocation Concealment | Blinding of participants and personnel | Blinding of outcome assessors | Incomplete outcome data | Selective reporting | Other Bias |
| Lee | Probably Low  (Randomization performed but method not described) | Probably low (Method of allocation concealment not documented but baseline characteristics are evenly distributed between control and intervention) | Probably low (blinding not described but objective outcome) | Probably low (Ethics section states that data was “treated anonymously during analysis”) | High (Greater than 10% dropout rate following randomization that was not accounted for during analysis - per-protocol analysis performed) | Probably high (no description of protocol publication, blood gas endpoints are reported but not outlined in methods) | Probably low (no funding or COI reported but study reporting unclear on methodology with randomization of patients performed but study described as an observational trial) |
| Cong | Probably Low  (Randomization planned but method not described) | Probably Low (Allocation concealment not documented, but baseline characteristics fairly even) | Probably low (Described as single blind but unlikely to apply to patients and personnel given intervention, however PO2 is an objective outcome) | Probably low (Described as single blind, statisticians most likely group to be blinded and PO2 is an objective outcome) | Probably low (Unknown if ITT or per-protocol analysis, but no reported loss to follow-up) | Probably low (No documented trial registration but protocol was approved by the local ethics committee) | Low (No funding or COI reported) |
| Doshi | Low (Computer generated randomization schedule) | Probably low  (Sealed, sequentially numbered, envelopes-did not mention if opaque) | Probably low (Patients and personnel not blinded however PO2 is an objective outcome) | Probably low (Did not describe if outcome assessors were blinded to intervention, however PO2 is an objective outcome) | Definitely no  (ITT analysis with pre-specified plan for analysis of crossovers, no losses) | Low (All pre-specified outcomes were reported and trial was registered) | Probably high (Vapotherm was involved with trial design, selection or sites, and site management) |
| Cortegiani | Low  (Computer generated randomization sequence) | Low  (Sequentially numbered, sealed, opaque envelopes) | Probably low (Patients and personnel not blinded however PO2 is an objective outcome) | Probably low (Research collectors were not blinded, however statisticians were blinded and PO2 is an objective outcome) | Probably low  (ITT and per-protocol analysis reported, very low loss to follow-up) | Low (All pre-specified outcomes reported, protocol published and trial registered on clinical trials.gov) | Low  (No funding, some device support from Fisher and Paykel however it was stated they played no role in protocol design, data management, interpretation or analysis) |
| Papachatzakis | Probably low  (Stated “Randomized”, but method not reported) | Probably low  (Method of allocation concealment not documented, but baseline characteristics fairly even) | Probably low (Not possible to blind NIV vs HFNC, however PCO2 is an objective outcome) | Probably low (Not discussed however PCO2 is an objective outcome) | Probably low (-Did not define if ITT, but no reported loss of patients) | Probably low (Did not describe trial registration, however a trial protocol was submitted a priori to the local research ethics board/REB) | Low  (No funding or COI reported) |
| **PCO2** | Random Sequence Generation | Allocation Concealment | Blinding of participants and personnel | Blinding of outcome assessors | Incomplete outcome data | Selective reporting | Other Bias |
| Lee | Probably Low  (Randomization performed but method not described) | Probably low (Method of allocation concealment not documented but baseline characteristics are evenly distributed between control and intervention) | Probably low (blinding not described but objective outcome) | Probably low (Ethics section states that data was “treated anonymously during analysis”) | High (Greater than 10% dropout rate following randomization that was not accounted for during analysis - per-protocol analysis performed) | Probably high (no description of protocol publication, blood gas endpoints are reported but not outlined in methods) | Probably low (no funding or COI reported but study reporting unclear on methodology with randomization of patients performed but study described as an observational trial) |
| Sklar | Probably low (Patients enrolled in random order but method of randomization not described) | Probably low (Opaque, sealed envelope but not sequential numbering not described) | Probably low (Patients and personnel not blinded, but PCO2 is an objective outcome) | Probably low (no blinding described, but PCO2 is an objective outcome) | Low  (Crossover trial with low loss to follow up in PCO2 outcome) | Low (Pre-specified outcomes including PCO2 reported, there were subjective outcomes added; trial registered on clinicaltrials.gov) | Probably high (There was industry funding to complete the study, role in trial design and implementation not described) |
| Papachatzakis | Probably low  (Stated “Randomized”, but method not reported) | Probably low  (Method of allocation concealment not documented, but baseline characteristics fairly even) | Probably low (Not possible to blind NIV vs HFNC, however PCO2 is an objective outcome) | Probably low (Not discussed however PCO2 is an objective outcome) | Probably low (Did not define if ITT, but no reported loss of patients) | Probably low (Did not describe trial registration, however a trial protocol was submitted a priori to the local research ethics board) | Low  (No funding or COI reported) |
| Cortegiani | Low  (Computer generated randomization sequence) | Low  (Sequentially numbered, sealed, opaque envelopes) | Probably low (Patients and personnel not blinded however PCO2 is an objective outcome) | Probably low (Research collectors were not blinded, however statisticians were blinded and PCO2 is an objective outcome) | Probably low  (ITT and per-protocol analysis reported, very low loss to follow-up) | Low (All pre-specified outcomes reported, protocol published and trial registered on clinical trials.gov) | Low  (No funding, some device support from Fisher and Paykel however it was stated they played no role in protocol design, data management, interpretation or analysis) |
| Cong | Probably Low  (Randomization planned but method not described) | Probably Low (Allocation concealment not documented, but baseline characteristics fairly even) | Probably low (Described as single blind but unlikely to apply to patients and personnel given intervention, however PCO2 is an objective outcome) | Probably low (Described as single blind, statisticians most likely group to be blinded and PCO2 is an objective outcome) | Probably low (Unknown if ITT or per-protocol analyses performed, but no reported loss to follow-up) | Probably low (No documented trial registration but protocol was approved by the local ethics committee) | Low (No funding or COI reported). |
| Doshi | Low (Computer generated randomization schedule) | Probably low  (Sealed, sequentially numbered, envelopes-did not mention if opaque) | Probably low (Patients and personnel not blinded however PCO2 is an objective outcome) | Probably low (Did not describe if outcome assessors were blinded to intervention, however PCO2 is an objective outcome) | Definitely no  (ITT analysis with pre-specified plan for analysis of crossovers, no loss to follow-up) | Low (All pre-specified outcomes were reported and trial was registered) | Probably high (Vapotherm was involved with trial design, selection or sites, and site management) |
| Rezaei | Low (Computer generated randomization) | Probably low (Allocation concealment not described; baseline characteristics fairly similar, baseline PaCO2 higher in group B but does not reach significance) | Probably low (Patients and personnel not blinded but PCO2 is an objective outcome) | Probably low (Investigators and data analyzers blinded and PCO2 is an objective outcome) | Probably low (Crossover trial, no losses to follow up) | Low (Pre-specified outcomes including PCO2 reported but more added from trial registration; trial registered on Iranian clinical trial registry) | Low (No COI or funding reported) |
| **Respiratory Rate** | Random Sequence Generation | Allocation Concealment | Blinding of participants and personnel | Blinding of outcome assessors | Incomplete outcome data | Selective reporting | Other Bias |
| Lee | Probably Low  (Randomization performed but method not described) | Probably low (Method of allocation concealment not documented but baseline characteristics are evenly distributed between control and intervention) | Probably low (blinding not described but objective outcome) | Probably low (Ethics section states that data was “treated anonymously during analysis”) | High (Greater than 10% dropout rate following randomization that was not accounted for during analysis - per-protocol analysis performed) | Probably high (no description of protocol publication, blood gas endpoints are reported but not outlined in methods) | Probably low (no funding or COI reported but study reporting unclear on methodology with randomization of patients performed but study described as an observational trial) |
| Doshi | Low (Computer generated randomization schedule) | Probably low  (Sealed, sequentially numbered, envelopes-did not mention if opaque) | Probably low (Patients and personnel not blinded however RR is an objective outcome) | Probably low (Did not describe if outcome assessors were blinded to intervention, however RR is an objective outcome) | Definitely no  (ITT analysis with pre-specified plan for analysis of crossovers, no losses) | Low (All pre-specified outcomes were reported and trial was registered) | Probably high (Vapotherm was involved with trial design, selection or sites, and site management) |
| Papachatzakis | Probably low  (Stated “Randomized”, but method not reported) | Probably low  (Method of allocation concealment not documented, but baseline characteristics fairly even) | Probably low (Not possible to blind NIV vs HFNC, however RR is an objective outcome) | Probably low (Not discussed however RR is an objective outcome) | Probably low (Did not define if ITT, but no reported loss of patients | Probably low (Did not describe trial registration, however a trial protocol was submitted a priori to REB) | Low  (No funding or COI reported) |
| Cortegiani | Low  (Computer generated randomization sequence) | Low  (Sequentially numbered, sealed, opaque envelopes) | Probably low (Patients and personnel not blinded however RR is an objective outcome) | Probably low (Research collectors were not blinded, however statisticians were blinded and RR is an objective outcome) | Probably low  (ITT and per-protocol analysis reported, very low loss to follow-up) | Low (All pre-specified outcomes reported, protocol published and trial registered on clinical trials.gov) | Low  (No funding, some device support from Fisher and Paykel however it was stated they played no role in protocol design, data management, interpretation or analysis) |
| Sklar | Probably low (Patients enrolled in random order but method of randomization not described) | Probably low (Opaque, sealed envelope but not sequential numbering not described) | Probably low (Patients and personnel not blinded, but RR is an objective outcome) | Probably low (No blinding described, but RR is an objective outcome) | Low  (Crossover trial with no loss to follow up in RR outcome) | Low (Pre-specified outcomes including RR reported, there were subjective outcomes added; trial registered on clinicaltrials.gov) | Probably high (There was industry funding to complete the study, role in trial design and implementation not described) |
| Rezaei | Low (Computer generated randomization) | Probably low (Allocation concealment not described; baseline characteristics fairly similar, baseline PaCO2 higher in group B but does not reach significance) | Probably low (Patients and personnel not blinded but RR is an objective outcome) | Probably low (States investigators and data analyzers blinded and RR is an objective outcome) | Probably low (Crossover trial, no losses to follow up) | High (Pre-specified outcomes reported but more added from trial registration; trial registered on Iranian clinical trial registry) | Low (No COI or funding reported) |


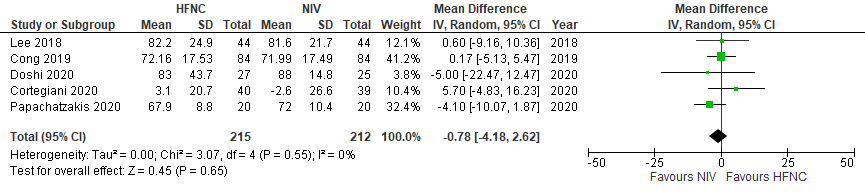


e-Figure 1 Forest plot of change in PaO_2_


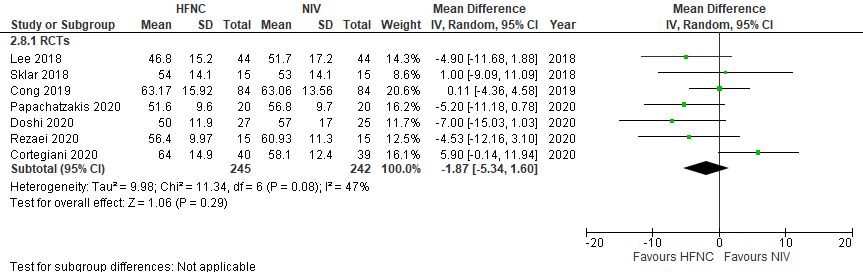


e-Figure 2 Forest plot of change in PCO_2_


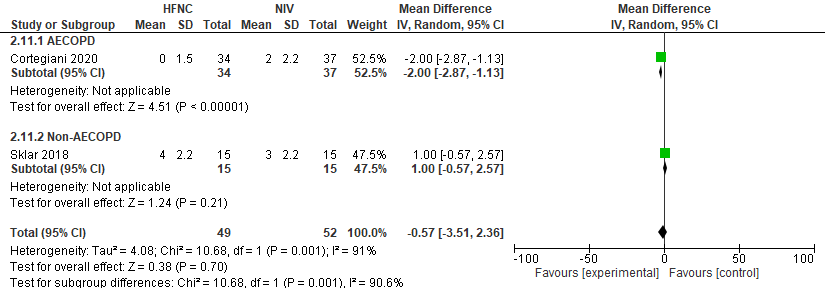


e-Figure 3 Forest plot of change in comfort - subgroup analysis by AECOPD studies alone


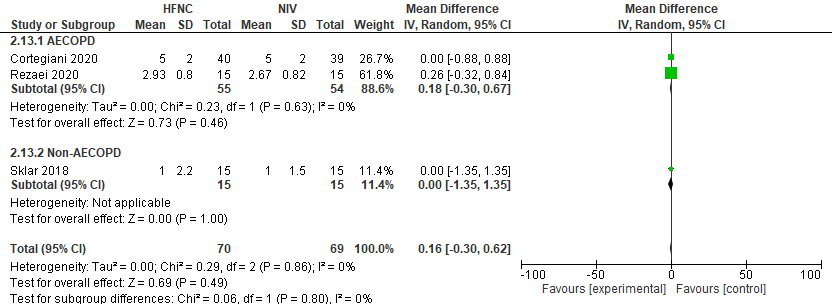


e-Figure 4 Forest plot of change in dyspnea - subgroup analysis by AECOPD studies alone


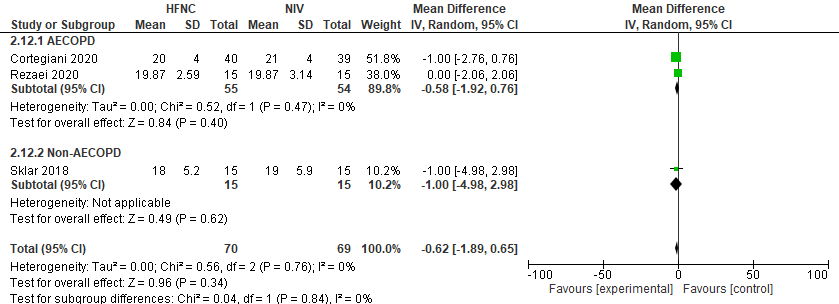


e-Figure 5 Forest plot of change in respiratory rate - subgroup analysis by AECOPD studies alone


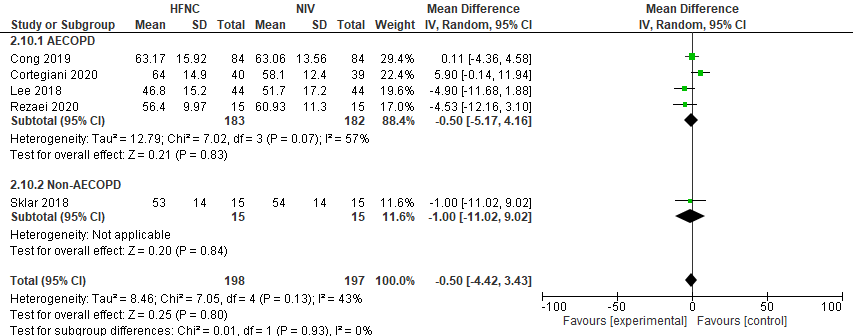


e-Figure 6 Forest plot of change in PaCO_2_ - subgroup analysis by AECOPD studies alone


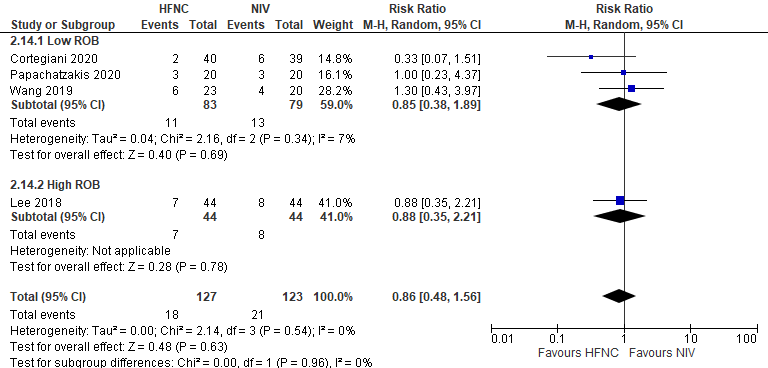


e-Figure 7 Forest plot of mortality - subgroup analysis by risk of bias


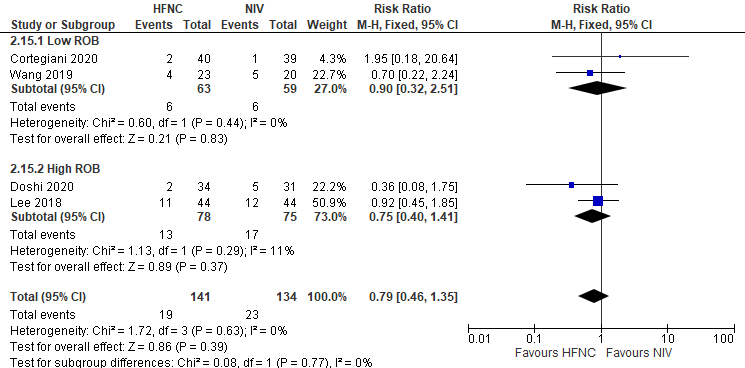


e-Figure 8 Forest plot of intubation - subgroup analysis by risk of bias


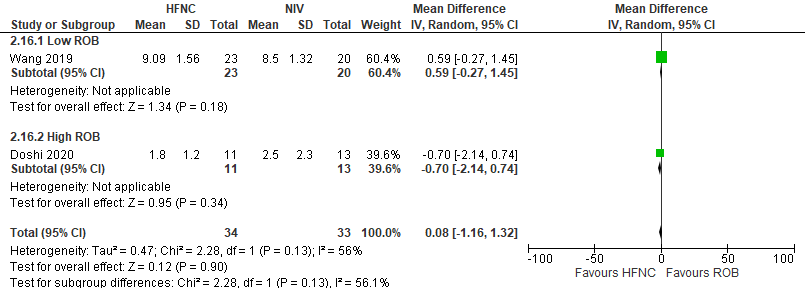


e-Figure 9 Forest plot of ICU Length of Stay - subgroup analysis by risk of bias

*
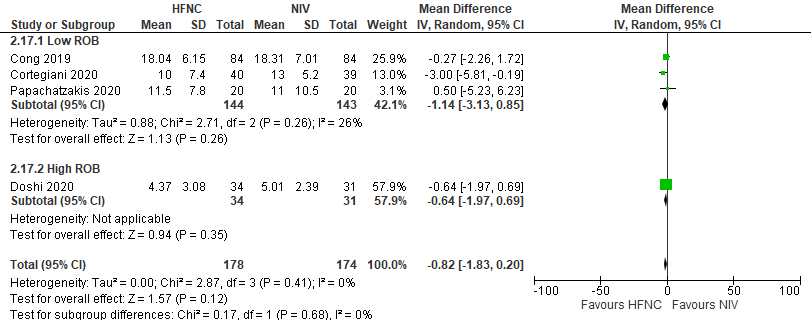
*

e-Figure 10 Forest plot of Hospital Length of Stay - subgroup analysis by risk of bias


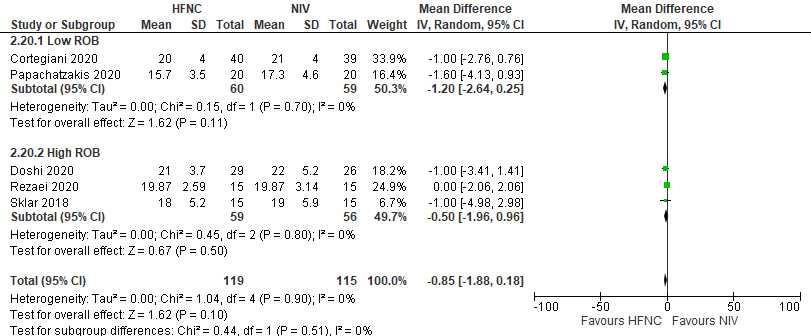


e-Figure 11 Forest plot of respiratory rate - subgroup analysis by risk of bias


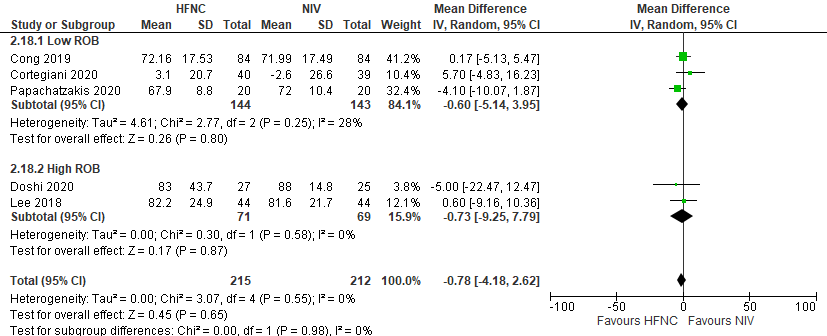


e-Figure 12 Forest plot of change in PaO_2_ - subgroup analysis by risk of bias


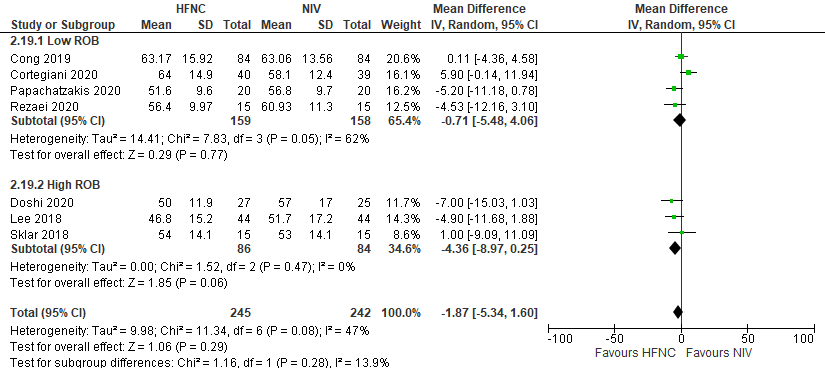


e-Figure 13 Forest plot of change in PaCO_2_ - subgroup analysis by risk of bias


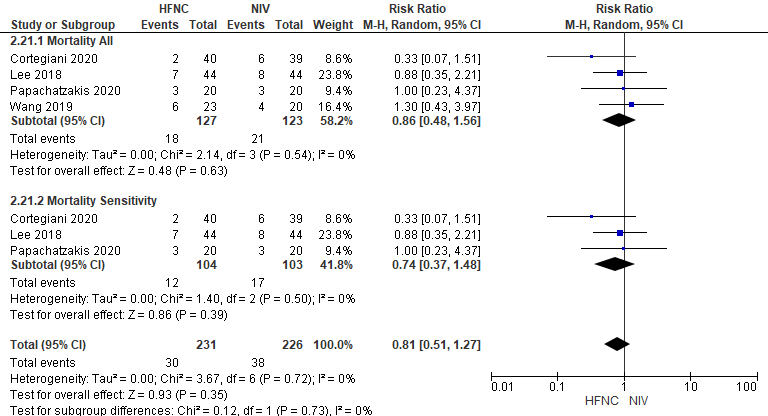


e- Figure 14 Forest plot of mortality - subgroup analysis excluding Wang et al.


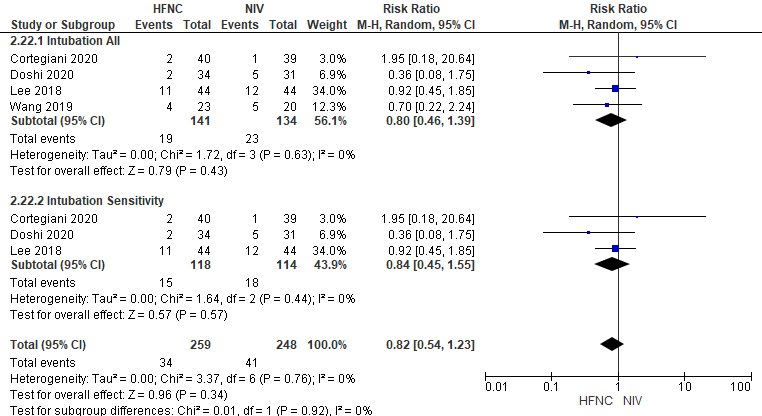


e-Figure 15 Forest plot of intubation - subgroup analysis excluding Wang et al.


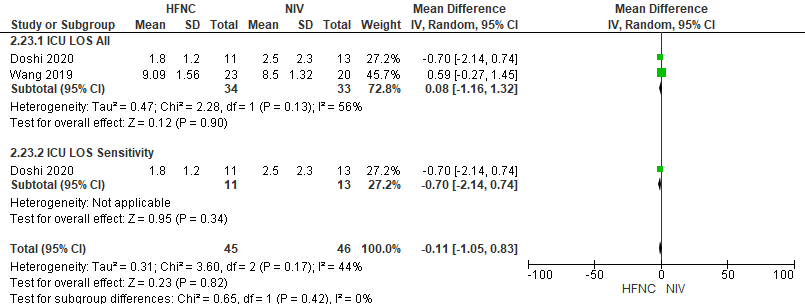


e-Figure 16 Forest plot of ICU Length of Stay - subgroup analysis excluding Wang et al


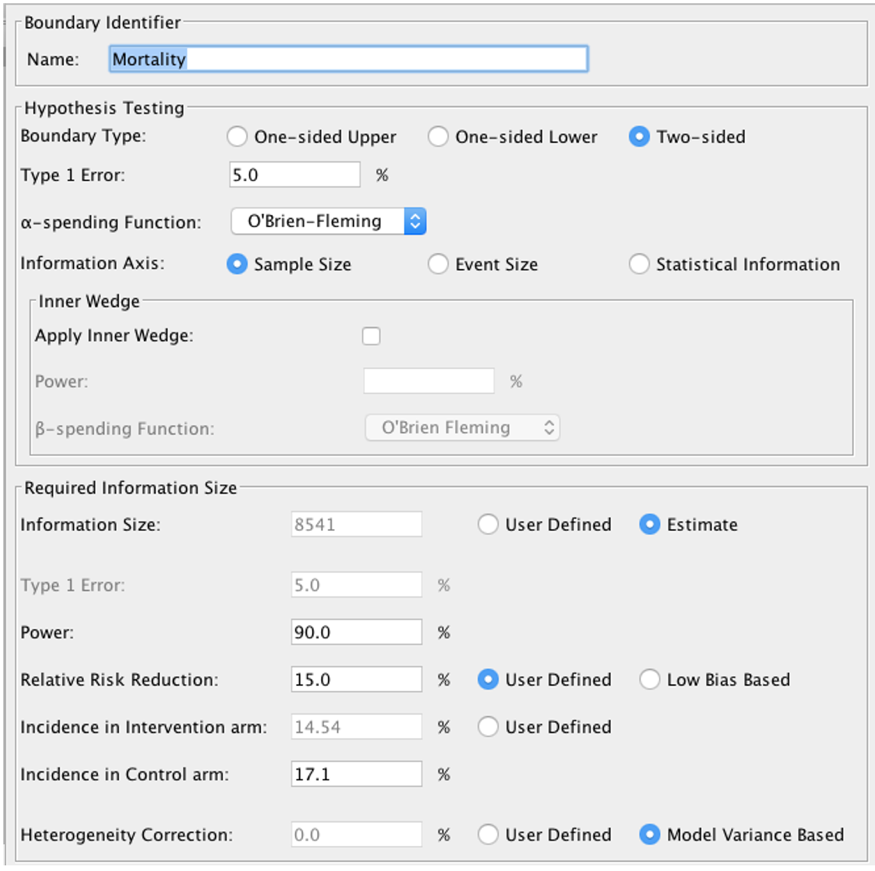


e-Figure 17: Trial sequential analysis for mortality


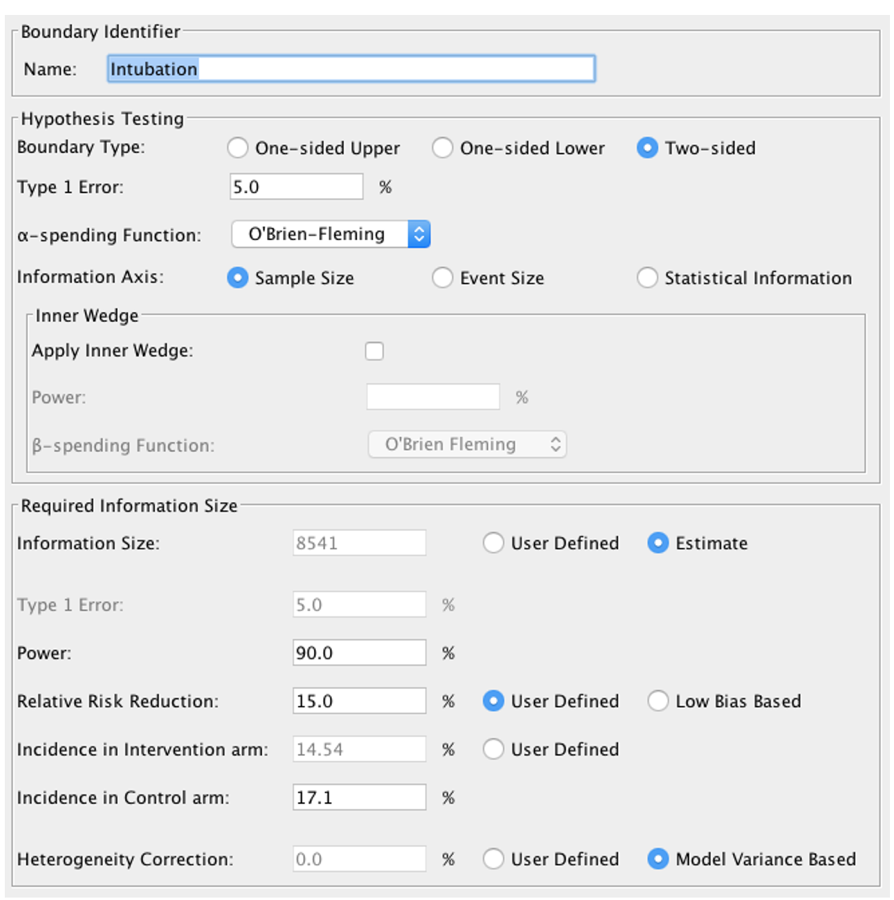


e-Figure 18: Trial sequential analysis for intubation


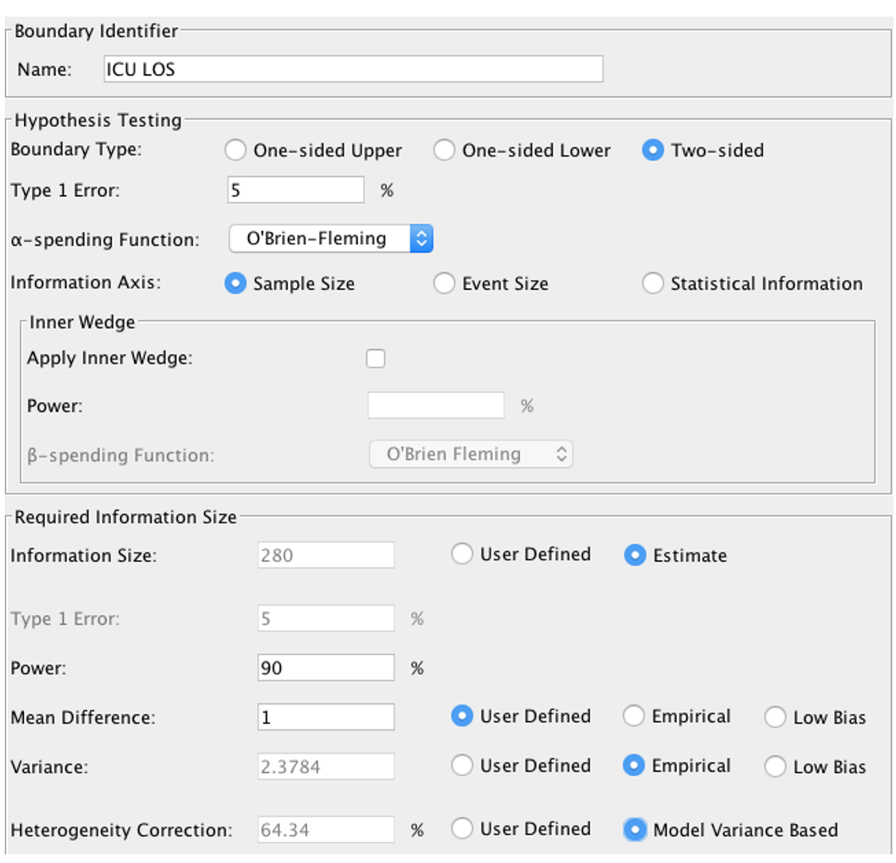


e-Figure 19: Trial sequential analysis for ICU length of stay
